# The IAA- and ABA-responsive transcription factor CgMYB58 upregulates lignin biosynthesis and triggers juice sac granulation in pummelo

**DOI:** 10.1038/s41438-020-00360-7

**Published:** 2020-09-01

**Authors:** Meiyan Shi, Xiao Liu, Haipeng Zhang, Zhenyu He, Hongbin Yang, Jiajing Chen, Jia Feng, Wenhui Yang, Youwu Jiang, Jia-Long Yao, Cecilia Hong Deng, Juan Xu

**Affiliations:** 1 Key Laboratory of Horticultural Plant Biology, Ministry of Education, Wuhan, Hubei 430070 China; 2 The New Zealand Institute for Plant and Food Research Limited, Private Bag 92169, Auckland, 1142 New Zealand

**Keywords:** Gene expression, Transcriptomics, Metabolomics, Transcriptional regulatory elements

## Abstract

In citrus, lignin overaccumulation in the juice sac results in granulation and an unpleasant fruit texture and taste. By integrating metabolic phenotyping and transcriptomic analyses, we found 702 differentially expressed genes (DEGs), including 24 transcription factors (TFs), to be significantly correlated with lignin content. *CgMYB58* was further identified as a critical R2R3 MYB TF involved in lignin overaccumulation owing to its high transcript levels in Huanong Red-fleshed pummelo (HR, *Citrus grandis*) fruits. Transient expression of *CgMYB58* led to an increase in the lignin content in the pummelo fruit mesocarp, whereas its stable overexpression significantly promoted lignin accumulation and upregulated 19 lignin biosynthetic genes. Among these genes, *CgPAL1*, *CgPAL2*, *Cg4CL1*, and *CgC3H* were directly modulated by CgMYB58 through interaction with their promoter regions. Moreover, we showed that juice sac granulation in pummelo fruits could be affected by indole-3-acetic acid (IAA) and abscisic acid (ABA) treatments. In HR pummelo, ABA significantly accelerated this granulation, whereas IAA effectively inhibited this process. Taken together, these results provide novel insight into the lignin accumulation mechanism in citrus fruits. We also revealed the theoretical basis via exogenous IAA application, which repressed the expression of *CgMYB58* and its target genes, thus alleviating juice sac granulation in orchards.

## Introduction

Lignin, which normally accumulates in secondary cell walls, has been reported to play a critical role in maintaining mechanical strength and facilitating the transport of water and nutrients in plants^1,2^, and is crucial for plant growth and responses to environmental stress^3,4^. In woody plants, a high lignin content in vascular bundles is a positive trait for woody products; however, lignin accumulation is undesirable in the fruit flesh of crop species, such as loquat (*Eriobotrya japonica*)^5,6^, pear (*Pyrus bretschneideri*)^7,8^, and citrus^9,10^. Therefore, understanding the regulatory mechanism underlying lignin biosynthesis and lignin accumulation is of great importance, and such knowledge could potentially be applied to assist fruit breeding programs.

In plants, the lignin biosynthetic pathway involves a series of enzymes, including phenylalanine ammonia lyase (PAL), cinnamate 4-hydroxylase (C4H), 4-coumarate CoA ligase (4CL), hydroxy cinnamoyl CoA, shikimate/quinate hydroxy cinnamoyl transferase (HCT), p-coumarate 3-hydroxylase (C3H), caffeoyl CoA 3-*O*-methyltransferase (CCoAOMT), cinnamoyl CoA reductase (CCR), caffeic acid *O*-methyltransferase, cinnamyl alcohol dehydrogenase, and ferulate 5-hydroxylase. Through reactions catalyzed by these enzymes, plants produce three types of lignin monomers, p-hydroxyphenyl (H) monomers, guaiacyl (G) monomers, and syringyl (S) monomers, which are then polymerized by laccases (LACs) or peroxidase^2,3,11^.

Transcription factors (TFs) regulating lignin biosynthetic genes have been well studied in various plant species, such as *Arabidopsis thaliana*^12^, *Populus trichocarpa*^13^, and switchgrass (*Panicum virgatum*)^14^. In *Arabidopsis*, AC elements bound specifically by MYBs are widely distributed in the promoters of *PAL1*, *PAL2*, *4CL1*, *4CL2*, *HCT*, *C3H1*, *CCoAOMT1*, *CCR1*, and *CAD5*^2^. AtMYB58 and AtMYB85 selectively bind AC elements in the promoter of *4CL1*, thereby regulating developmental lignification in vascular tissues^15,16^. Evidence has confirmed that MYBs can regulate lignin biosynthetic genes involved in secondary cell wall formation, including PvMYB58/63, PvMYB42/85, and PvMYB4, in switchgrass^14^. EjMYB1, a homolog of AtMYB58 in loquat, has been reported to modulate lignin biosynthesis during fruit storage^5^. In the stone cells of pear fruits, PbrMYB169 was found to upregulate lignification by activating the promoters of lignin biosynthetic genes^7^. In addition to MYBs, other TFs have also been revealed to play important roles in fruit lignin biosynthesis; e.g., EjNAC1, EjAP2-1, and EjHSF3 are involved in lignin formation in loquat^6,17,18^.

Citrus fruits, which are the most popular fruits worldwide, provide an assortment of functional components that are beneficial to human health, including flavonoids^19,20^, volatile compounds^21^, alkaloids^22^, and carotenoids^23–26^. However, in citrus fruit juice sacs, overaccumulation of lignin (a physiological disorder known as granulation) often occurs^9,10,27,28^. This granulation causes a “gritty” texture and dryness of the juice sacs, reduces consumer acceptance of the fruits, and is a severe problem in sweet orange (*Citrus sinensis*)^10^, Ponkan mandarin (*C. reticulata*)^28^ and Guanximiyou pummelo (*C. grandis*)^9^. Granulation is affected by disorders involving mineral nutrition, phytohormones, growth temperature, and genetic factors^29^. Previous findings have revealed that TFs play a crucial role in sweet orange granulation. For example, CsMYB330 and CsMYB85, which are homologs of AtMYB58/AtMYB63 and AtMYB85, respectively, have been reported to interact with CsMYB308 through the binding of the *Cs4CL1* promoter^10,30^ to regulate juice sac granulation.

Among citrus fruits, pummelo fruits are especially prone to granulation during ripening and storage^9^. However, our knowledge of the regulation of lignin accumulation and thus granulation is limited in pummelo fruits. In the present study, we investigated juice sac granulation and evaluated lignin accumulation in four pummelo cultivars, Huanong Red-fleshed (HR), Hirado Buntan (HB), Fenghuang (FH), and Kao Pan (KP), throughout different fruit developmental stages. We found that indole-3-acetic acid (IAA)- and abscisic acid (ABA)-responsive CgMYB58 played a critical role in lignin biosynthesis. This TF can interact with the promoter regions of *CgPAL1*, *CgPAL2*, *Cg4CL1*, and *CgC3H*. These results contribute to our understanding of the genetic mechanism controlling juice sac granulation and provide a potential practical approach to inhibiting this phenomenon in citrus orchards.

## Results

### Lignin excessively overaccumulated during juice sac granulation in pummelo

**Fig. 1:**
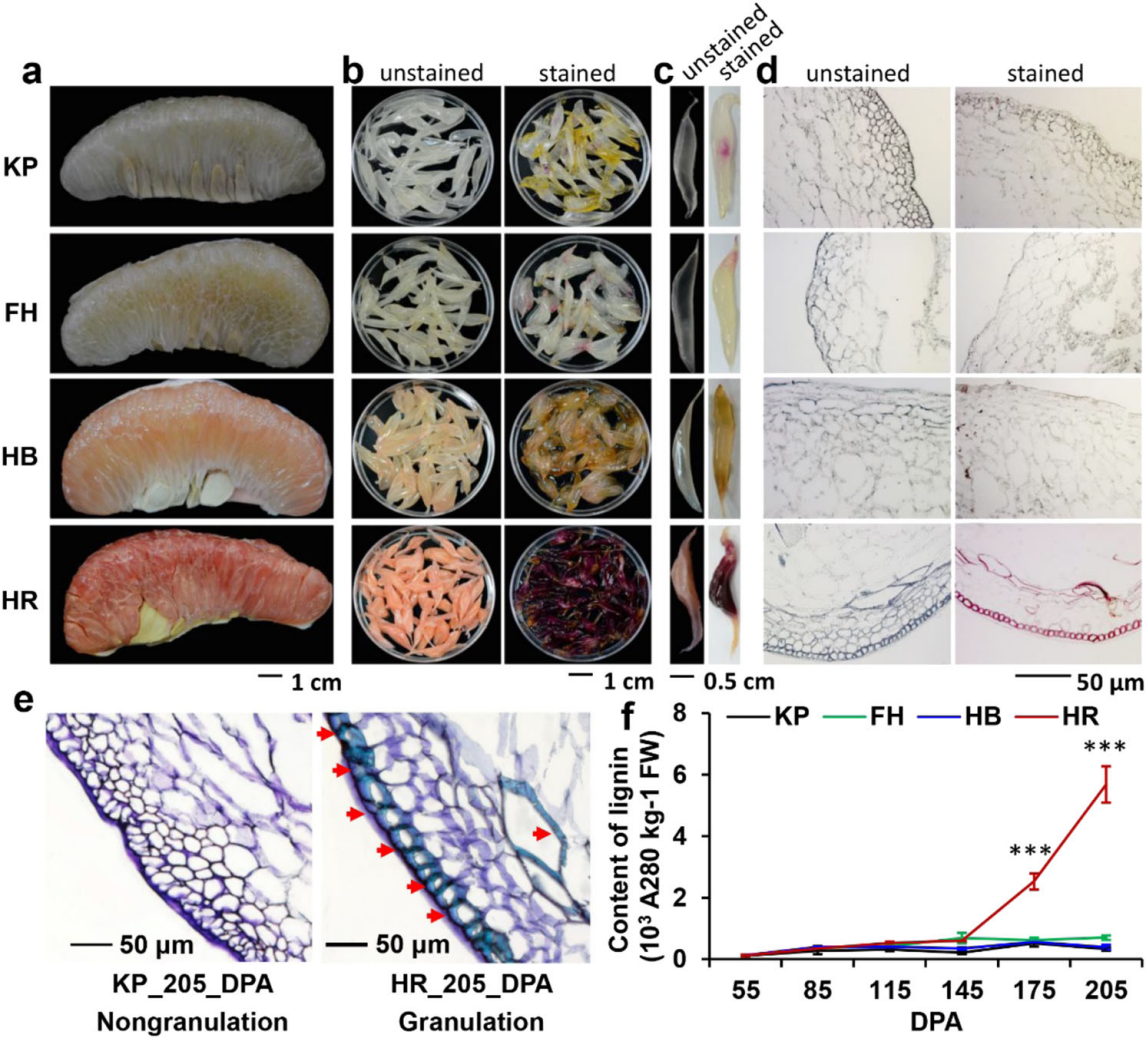
HR fruits accumulated high lignin content in their juice sacs at late developmental stages. **a** The juice sacs of four pummelo cultivars, Kao Pan (KP), Fenghuang (FH), Hirado Buntan (HB), and Huanong Red-fleshed (HR), were collected from mature fruits at 205 DPA. **b** The juice sacs were separated (left) and stained with phloroglucinol hydrochloride dye (right). The high lignin contents in the HR juice sacs resulted in a dark red color (**b**, **c**). **d** The HR juice sacs were stained dark red and their sections were stained red, indicating a relatively high level of lignin accumulation. The bar applies to all the images in the same panel. **e** Paraffin sections of KP and HR juice sacs were stained with toluidine blue. The arrowheads point to blue-stained thickened cell walls with a high lignin content. **f** The lignin content in the juice sacs of four pummelo cultivars was measured at six developmental stages. In HR, the lignin content increased significantly and accumulated much more than that in the other cultivars after 145 DPA. The asterisks indicate significant differences in HR compared with KP, FH, and HB at 175 and 205 DPA, and were analyzed by Duncan’s multiple comparison test (***P < 0.05)

We investigated juice sac granulation in four pummelo cultivars, HR, HB, FH, and KP, at 205 days post anthesis (DPA) and found that HR exhibited the most severe granulation (Fig. 1a). Moreover, the granulated juice sacs of HR were stained deep red with the lignin-specific stain phloroglucinol-HCl, whereas the other three pummelo cultivar fruits showed only very small red spots or were colorless after staining (Fig. 1b, c). The subsequent examination of paraffin-embedded sections showed that the cell walls of the HR juice sacs were stained red and purple by phloroglucinol-HCl and toluidine blue, respectively, and were thickened, owing to the excessive accumulation of lignin (Fig. 1d, e).

Next, we measured the lignin contents of the juice sacs of these four cultivars at 55, 85, 115, 145, 175, and 205 DPA (collected in 2016). The lignin content of the juice sacs gradually increased in HR during all developmental stages and the lignin content in HR was much higher than that in KP, FH, and HB at 175 and 205 DPA (Fig. 1f). The lignin accumulation in the juice sacs of HR sharply increased from 145 to 205 DPA and reached 5.69 × 10^3^ A280 kg^−1^ at 205 DPA (Fig. 1f). Moreover, we assessed the lignin contents of nine pummelo cultivars at ~200 DPA (collected in 2014). The data showed that HR contained the highest lignin content among all tested cultivars, whereas the others maintained lower levels of lignin, with an average of 0.6 × 10^3^ A280 kg^−1^ (Supplementary Fig. S1). Highly significant lignin accumulation was also observed in San Hong (a bud mutant of Guanximiyou) (Supplementary Fig. S2a, b), which has been reported to readily undergo juice sac granulation^9^. Taken together, these results indicated that the juice sac granulation of pummelo resulted from the overaccumulation of lignin.

### Transcriptomic and qRT-PCR analyses revealed that CgMYB58 modulates lignin biosynthetic genes

To explore the mechanism of granulation in HR, we performed transcriptomic analyses using the juice sacs of HR and two controls (HB and KP) at 55, 85, 115, 145, and 205 DPA. The RNA sequencing (RNA-seq) approach produced ~6 Gb of data for each sample and ~92% of the reads were mapped to the pummelo genome^31^ (Supplementary Table S1). Principal component analysis of fragments per kilobase of transcript per million mapped reads (FPKMs) and differentially expressed gene (DEG) analyses were performed (Supplementary Fig. S3), allowing us to further analyze DEGs enriched in the biosynthesis of secondary metabolites (Supplementary Fig. S4a) and phenylpropanoid metabolites (Supplementary Fig. S4b) by KEGG (Kyoto Encyclopedia of Genes and Genomes) analysis.

**Fig. 2:**
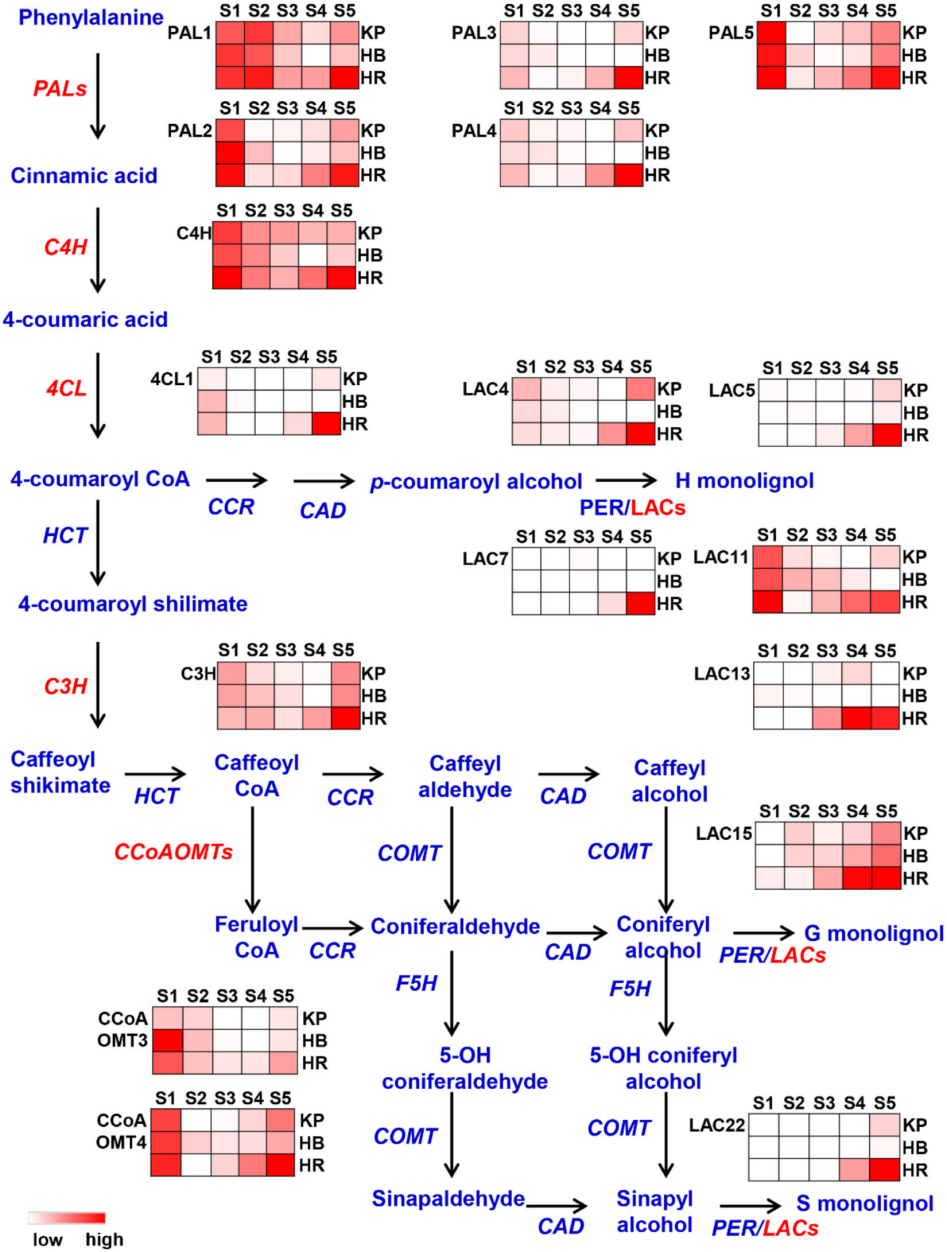
Transcriptome analyses of lignin biosynthetic genes. Heatmap of transcript levels in FPKM for genes that are involved in the lignin biosynthetic pathway and are distinctly expressed during five developmental stages between three cultivars. S1 to S5 represent 55, 85, 115, 145, and 205 DPA, respectively. The darker red color indicates a higher expression of the gene. The pale color shows a lower or lack of expression

Owing to the lignin content being greatly different between HR and the other pummelo cultivars, the FPKMs of lignin biosynthetic genes were analyzed (Fig. 2 and Supplementary Table S2). The transcript levels of the following genes sharply increased from 145 DPA to 205 DPA in HR: *CgPAL1*, *−2*, *−3*, *−4*, and *−5*; *CgC4H*; *Cg4CL1*; *CgC3H*; *CgCCoAOMT3* and *−4*; and *CgLAC4*, *−5*, *−7*, *−11*, *−13*, *−15*, and *−22* (Fig. 2). The real-time quantitative reverse-transcription PCR (qRT-PCR) results for *CgPAL1*, *CgPAL2*, *Cg4CL1*, and *CgC3H* were consistent with the FPKM results (Supplementary Fig. S5 and Supplementary Table S2).

**Fig. 3:**
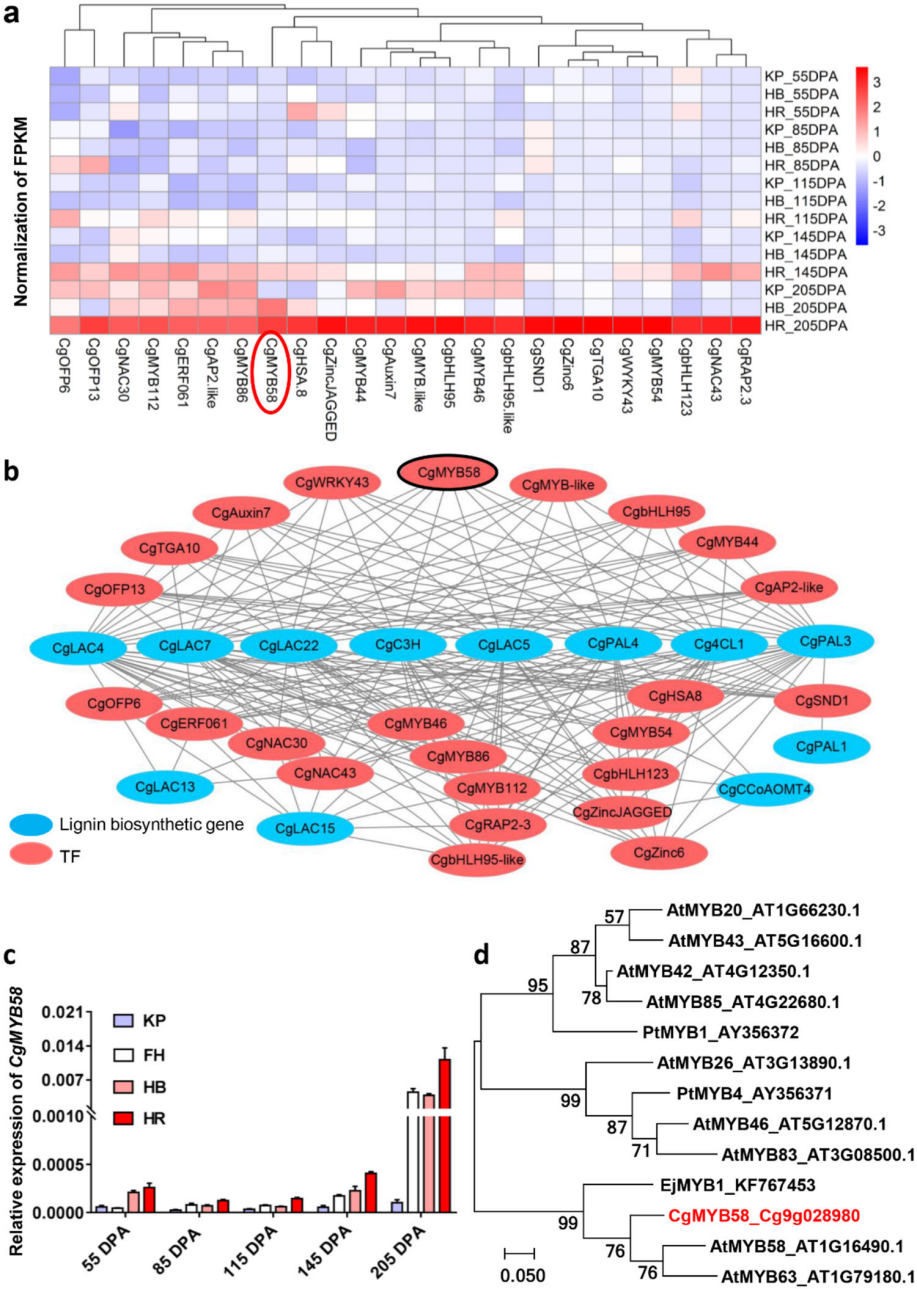
Transcriptome analyses of associated transcription factors. **a** Heatmap of transcription factors (TFs) involved in lignin biosynthesis. **b** Gene expression networks showing TFs (potential regulators) and genes encoding lignin biosynthetic enzymes. The black lines indicate that the coefficient value between the node genes is higher than 0.8. **c** Expression level of *CgMYB58* in KP, FH, HB, and HR at 55, 85, 115, 145, and 205 DPA. DPA: Days post anthesis. The housekeeping gene used was described in a previous report^26^. **d** Phylogenetic analysis of CgMYB58 and related proteins from other plant species. The scale bar represents 0.05 substitutions per site. The red color indicates CgMYB58

According to the juice sac granulation phenotype and lignin accumulation pattern (Fig. 1), we then selected the total lignin content as a bait for assessing the correlation with genes from the transcriptome. The genes correlated with the total lignin content with an absolute coefficient ≥ 0.75 were chosen as candidates. A total of 702 genes were found to be related to the total lignin content (Supplementary Table S3). According to GO enrichment analysis, the genes corresponding to lignin content were significantly enriched in the phenylpropanoid pathway and the lignin catabolic/metabolic pathway (Supplementary Fig. S6). Among these genes, 24 TFs were identified, which were abundant in transcripts in HR at 145 DPA and 205 DPA (Fig. 3a), and exhibited a relatively strong correlation (coefficient ≥ 0.8) with 12 lignin biosynthetic genes (Fig. 3b). The qRT-PCR results for *CgMYB46*, *CgMYB44*, *CgMYB112*, and *CgbHLH95* were also consistent with the FPKM results (Supplementary Fig. S5 and Supplementary Table S2), and the expression pattern of these TFs that were highly expressed in HR at the examined stages was in agreement with the high lignin content. Remarkably, homologous genes of *CgMYB58*, *CgMYB46*, and *CgSND1* have been previously reported to modulate secondary cell wall synthesis in *Arabidopsis*^15,16,32^. We showed that *CgMYB58* exhibited a higher transcript level than did *CgMYB46* and *CgSND1* in HR at 205 DPA (Supplementary Table S2). Our qRT-PCR assay revealed that *CgMYB58* was significantly highly expressed in HR compared with KP, FH, and HB throughout all developmental stages (Fig. 3c). In addition, *CgMYB58* was also highly expressed in San Hong granulated juice sacs and Guanxi red-fleshed pummelo in association with granulation and high *Cg4CL1* expression (Supplementary Fig. S2b-e). CgMYB58 was closely related to AtMYB58 and EjMYB1, as confirmed by phylogenetic analysis (Fig. 3d) and amino acid sequence alignment (Supplementary Fig. S7a); these proteins have been previously shown to regulate lignin biosynthesis in *Arabidopsis*^16^ and loquat^5^. Subcellular localization analysis revealed that CgMYB58 localized to the nucleus of citrus leaf protoplast cells (Supplementary Fig. S7b). Overall, we found that 702 DEGs were correlated with lignin accumulation, and that CgMYB58 was probably the key TF involved in lignin biosynthesis in pummelo fruits.

### Overexpression of *CgMYB58* promoted the accumulation of lignin in citrus tissues

The coding sequence of *CgMYB58* was identical among HR and other low-lignin-accumulating pummelo cultivars and thus was named *CgMYB58-1* (Supplementary Table S4). Therefore, in the following functional verification experiments, *CgMYB58-1* was used, as it is the dominant transcript expressed in HR fruit. To simplify, we replaced *CgMYB58-1* with *CgMYB58*. In addition, the promoter sequences of *CgMYB58* were mainly conserved among the pummelo cultivars but differed from those of sweet orange, lime (*C. aurantifolia*), and mandarin by two indels (Supplementary Table S4).

**Fig. 4:**
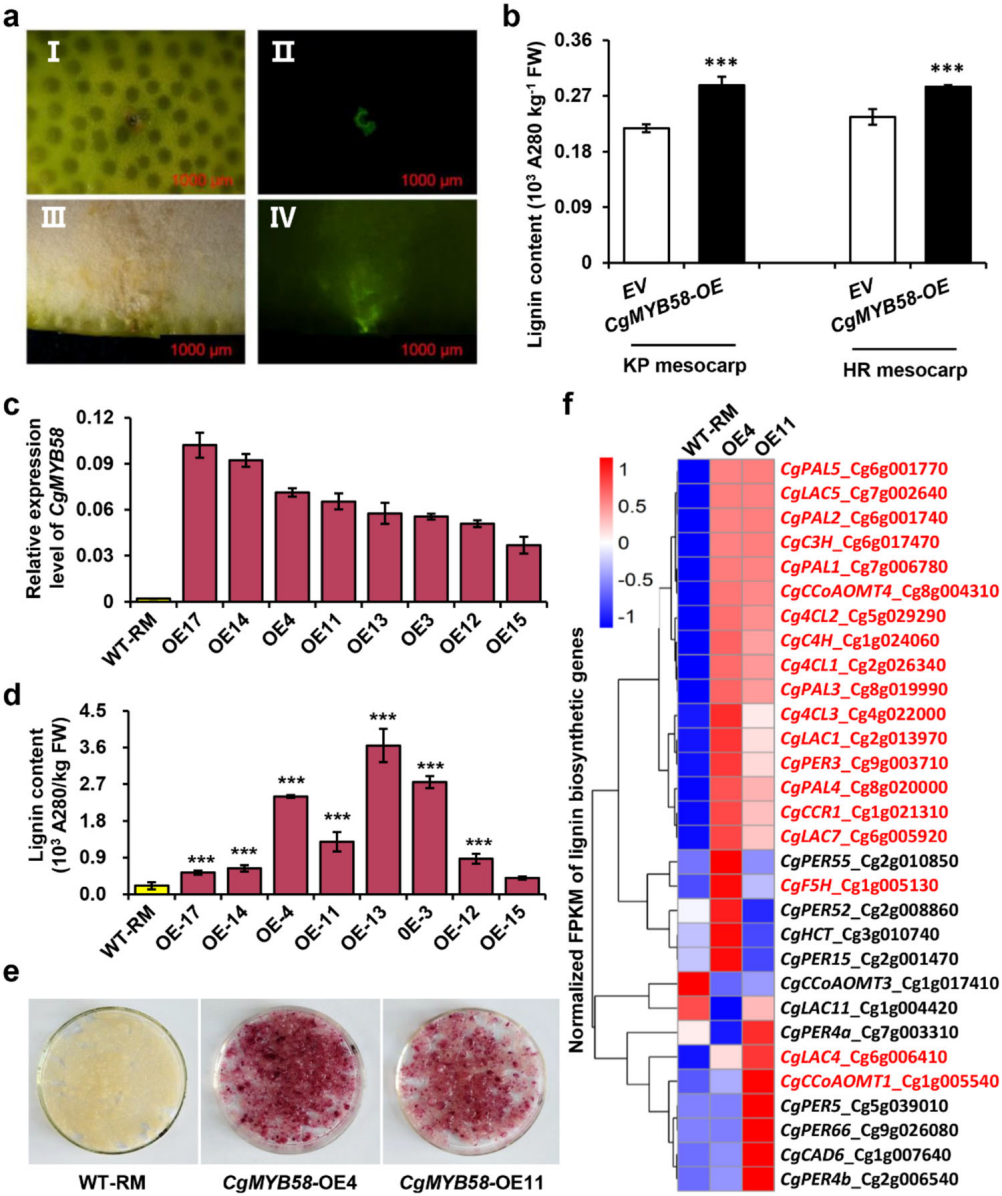
Overexpression of *CgMYB58* increased lignin accumulation and promoted the expression of lignin biosynthetic genes in citrus calli. **a** *CgMYB58* transiently expressed in pummelo mesocarp. HR fruit mesocarp were injected with *Agrobacteria* containing an empty vector (I, II) or a *CgMYB58* overexpression vector (III, IV) and imaged under bright-field (I, III) and GFP fluorescence conditions (II, IV). GFP fluorescence indicated that the vectors were successfully transferred into the pummelo fruit mesocarp. **b** Lignin contents were measured in pummelo fruit mesocarp tissue after transient transformation with an empty vector (EV) or a *CgMYB58* overexpression vector. **c** Relative expression levels of *CgMYB58* in WT-RM (Red Marsh) and eight transgenic RM calli lines overexpressing *CgMYB58*. **d** Lignin content in the calli of WT and eight transgenic lines. The asterisks indicate significant differences relative to WT-RM according to a *t*-test (***P < 0.05). **e** Phenotypes WT-RM calli and the two transgenic lines stained with phloroglucinol-HCl. The WT-RM calli did not show any staining, whereas the calli of the two transgenic RM lines (*CgMYB58*-OE04, -OE11) were stained red. **f** Cluster heatmap based on the transcript level of lignin biosynthetic genes. Among the 30 examined lignin biosynthetic genes, 19 were upregulated in the *CgMYB58*-OE lines, which are highlighted in red

To verify the biological function of *CgMYB58* in lignin accumulation, we first transiently expressed *CgMYB58* in the mesocarp of KP and HR fruits. The results emphasized that *CgMYB58* promoted lignin accumulation in the mesocarp of both pummelo cultivars (Fig. 4a, b). Wild-type (WT) Red Marsh (RM) calli were stably transformed with a *CgMYB58* overexpression (*CgMYB58*-OE) construct. The combined analyses of the levels of *CgMYB58* expression and lignin contents in eight *CgMYB58*-OE lines (Fig. 4c) indicated that, compared with the WT, seven of these lines exhibited a significantly higher lignin content (Fig. 4d). Furthermore, they were stained with phloroglucinol-HCl; the selected *CgMYB58*-OE calli lines displayed a deep red color, whereas the WT calli remained pale yellow (Fig. 4e). RNA-seq analysis was also carried out using two stable *CgMYB58*-OE lines and WT-RM calli, with three replicates analyzed for each sample. A total of 397 genes were found to be upregulated in *CgMYB58-*OE4 and *CgMYB58-*OE11 compared to the WT, whereas 372 were downregulated (Supplementary Fig. S8). The transcript levels of most genes involved in the lignin biosynthetic pathway were greatly altered (Fig. 4f), with 19 significantly increased in the *CgMYB58*-OE lines (Fig. 4f and Supplementary Table S5). Collectively, these data showed that overexpression of *CgMYB58* was responsible for lignin accumulation in citrus.

### CgMYB58 modulated lignin accumulation through interactions with the promoters of four lignin biosynthetic genes

**Fig. 5:**
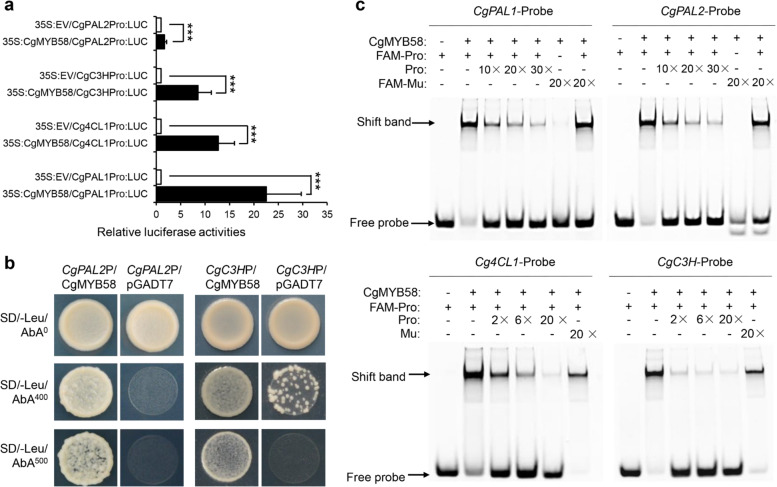
CgMYB58 bound to and transactivated the promoters of four lignin biosynthetic genes. **a** CgMYB58 increased the activities of the promoters of *CgPAL1*, *CgPAL2*, *Cg4CL1*, and *CgC3H* according to a dual LUC assay. LUC: luciferase. Pro: promoter; EV: empty vector; the asterisks indicate significant differences between the *CgMYB58* effector and empty vectors according to a *t*-test (***P < 0.05). **b** CgMYB58 could interact with the promoters of *CgPAL1* and *CgC3H* according to yeast one-hybrid assays. AbA: aureobasidin A; SD/-Leu: SD plate lacking leucine; P: promoter; a pGADT7 empty vector was used as negative control. **c** CgMYB58 directly bound to the AC elements in the four promoters according to EMSAs. EMSAs: electrophoretic mobility shift assays; FAM: 5’-FAM-labeled oligonucleotide; Pro: probe; Mu: mutant probe

To verify whether CgMYB58 could regulate lignin biosynthetic genes in citrus, the promoters of *CgPAL1*, *CgPAL2*, *Cg4CL1*, and *CgC3H* were cloned and sequenced. AC elements (5′-ACCAA(T)CC-3′ or 5′-ACCTAAC-3′) were identified in the promoter sequences at −383, −248, and −210 bp from the translational start codon for *CgPAL1*; at −500 and −295 bp for *CgPAL2*; at −224 bp for *Cg4CL1*; and at −1966, −1954, −1415, −457, −405, and −15 bp for *CgC3H* (Supplementary Table S6). To determine whether CgMYB58 could bind to these promoters, a dual luciferase (LUC) transcriptional activity assay was performed in tobacco leaves. A 35S:*CgMYB58* construct and an empty vector were used as effectors, whereas the promoters of *CgPAL1*, *CgPAL2*, *Cg4CL1*, and *CgC3H* were fused as reporters. The relative LUC activity derived from these promoters was strongly stimulated by CgMYB58 (Fig. 5a). Yeast one-hybrid assays (Y1Hs) then revealed that CgMYB58 could interact with the promoters of *CgPAL2* and *CgC3H* (Fig. 5b). Notably, the promoter of *Cs4CL1* of sweet orange was also bound by CsMYB330, a homolog of CgMYB58, as shown in a previous Y1H assay^10^. As the promoter sequences of *4CL1* and the coding sequence of *MYB58* were conserved between sweet orange and pummelo, the results were identical when using gene sequences from pummelo and thus were not presented here. Finally, using a pMAL-c5x-CgMYB58 fusion protein, we performed an electrophoretic mobility shift assay (EMSA) to verify these interactions. The purified pMAL-c5x-CgMYB58 protein could directly bind to the 25–27 bp probes containing the AC elements in the promoters of *CgPAL1*, *CgPAL2*, *Cg4CL1*, and *CgC3H* (Fig. 5c). These results demonstrated that CgMYB58 promoted the lignification process by directly upregulating the expression of four monolignol biosynthetic genes: *CgPAL1*, *CgPAL2*, *Cg4CL1*, and *CgC3H*.

### Lignin accumulation in response to IAA and ABA treatments

**Fig. 6:**
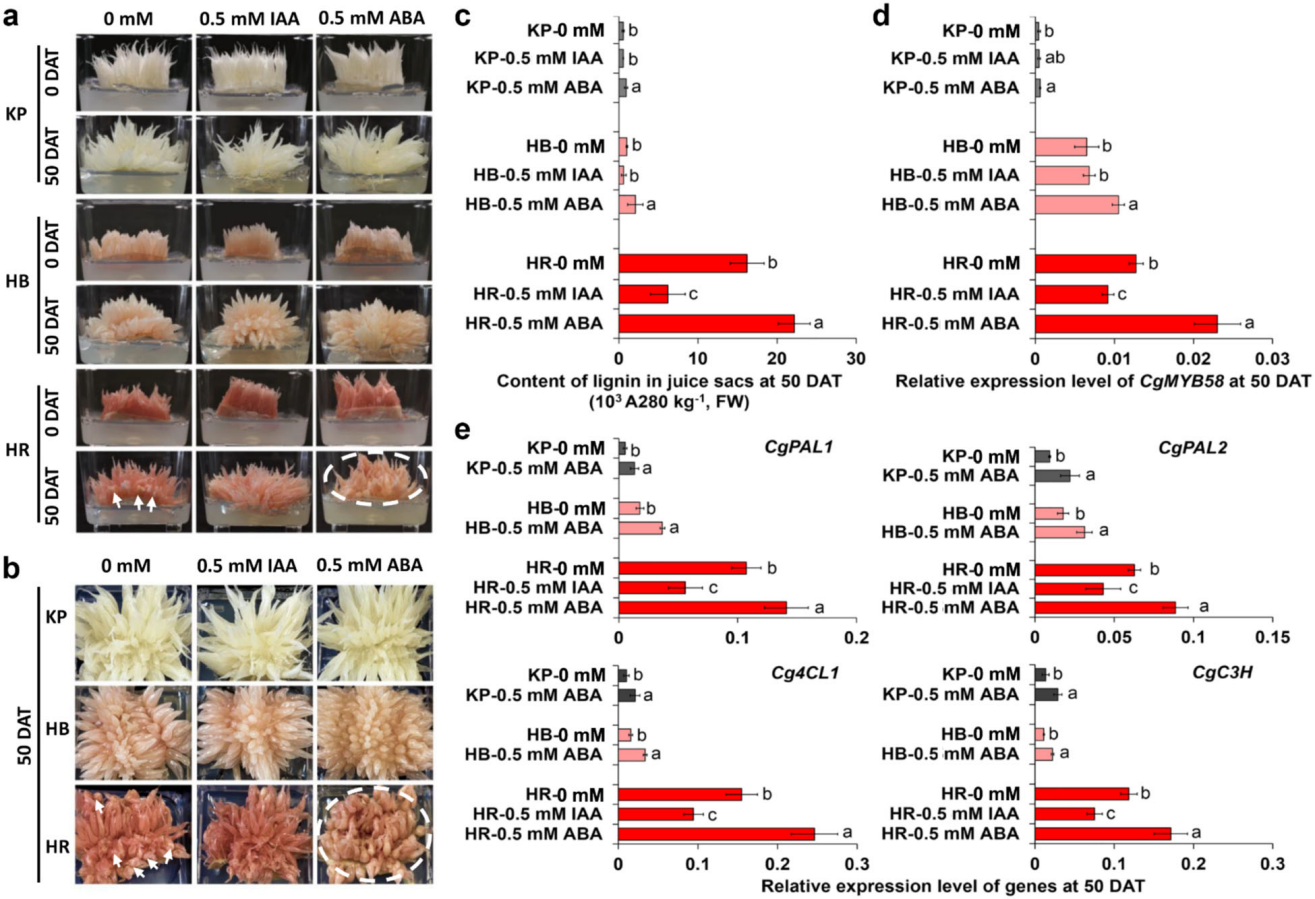
Juice sac granulation can be affected by ABA and IAA treatments through the regulation of *CgMYB58* and its targets in pummelo fruits. **a** Juice sacs of KP, HB, and HR pummelo fruits at 145 DPA were treated with phytohormones at 0 mM, 0.5 mM IAA, or 0.5 mM ABA, and were imaged at 0 and 50 days after treatment (DAT). **b** HR juice sacs were granulated in the 0 mM treatment at 50 DAT (highlighted by the white arrowhead) and granulation was not observed in the 0.5 IAA treatment but was increased in the 0.5 mM ABA treatment (highlighted by a dotted circle). KP and HB, used as controls, did not show granulation in any treatment. **c** Lignin content in juice sacs at 50 DAT. **d** Relative expression level of *CgMYB58* in juice sacs at 50 DAT. **e** Relative expression of lignin biosynthetic genes as revealed by qRT-PCR. The lowercase letters above the columns indicate significant differences. The data in (**c**) and (**d**), and the data for HR in (**e**) were analyzed by Duncan’s multiple comparison test (*P* < 0.05). The data for KP and HB (**e**) were analyzed according to a *t*-test (*P* < 0.05). IAA, indole-3-acetic acid. ABA, abscisic acid

The involvement of IAA and ABA in the lignification process has been investigated previously in horticultural plants^33,34^. In addition, we identified ABA-responsive elements in the promoters of *CgMYB58*, *CgPAL1*, *CgPAL2*, and *Cg4CL1*, whereas an auxin-responsive element was identified in the promoter of *CgC3H* (Supplementary Fig. S9). This raised the question of whether IAA and ABA could affect lignin accumulation in pummelo. Hence, we applied IAA and ABA treatments to KP, HB, and HR juice sacs during 50 days of continuous culture (Fig. 6a, b). Under IAA treatment, we measured a decrease in the lignin content in the HR juice sacs, whereas under ABA treatment, the lignin content increased and visible granulation was observed in the HR juice sacs (Fig. 6a-c). The expression levels of *CgMYB58* and its downstream genes were subsequently assessed using qRT-PCR (Fig. 6d, e). Taken together, the results showed that IAA treatment of juice sacs repressed the expression of *CgMYB58* and its target genes related to lignin biosynthesis, but ABA treatment promoted their expression, suggesting that *CgMYB58* responds to IAA and ABA in the regulation of the lignification process in citrus.

## Discussion

Previous evidence from the commercial pummelo cultivar Guanximiyou has shown that granulation due to excessive lignin accumulation can greatly damage fruit sensory quality during the post-harvest process^9^. We therefore investigated the correlation between juice sac granulation and lignin overaccumulation in pummelo. We found that severe granulation occurred in HR juice sacs, which contained higher levels of lignin in comparison with those of other pummelo cultivars. It is likely to be that red- and non-red-fleshed citrus fruit undergo lignification during long-term storage^9,10^ or in response to stress related to light, water, and temperature. However, under normal physiological conditions, our study showed that a high expression level of *CgMYB58* in HR was involved in lignin accumulation leading to granulation.

Lignin accumulation is crucial for plant developmental cues and responses to environmental stress in loquat fruit^6^, *Populus*^35^, and cotton (*Gossypium* spp.)^36^. Several types of TFs, such as MYBs, NACs, and ERFs, have been reported to regulate lignin biosynthesis genes in plants^6,12,13^. In *Arabidopsis*, AtMYB58 and AtMYB85 selectively bind to AC elements to regulate lignification in vascular tissues^15,16^. In this study, by integrating transcriptome and metabolite analyses, we found that CgMYB58 promoted the expression of four upstream core monolignol biosynthetic genes by directly binding to AC elements in the promoters of these genes. A homolog of *CgMYB58* in sweet orange, *CsMYB330*, was also identified as being abundant in granulated flesh, together with its target gene *Cs4CL*^10^. Our LUC assays and EMSAs showed that CgMYB58 could regulate not only *Cg4CL1* but also three other genes (*CgPAL1*, *CgPAL2*, and *CgC3H*) involved in lignin biosynthesis (Fig. 4). Notably, the *PAL1*, *PAL2*, and *C3H* genes have never been reported to be directly regulated by MYB58 in horticultural species. Moreover, CgMYB58 also upregulated downstream genes, such as *LAC1*, *−4*, *−5*, and *−7* (Fig. 4 and Supplementary Table S5), suggesting that its function is broader than that of lignin biosynthetic genes than was previously thought^10^. Interestingly, *CgMYB58* was also highly expressed in granulated San Hong juice sacs and in Guanxi red-fleshed pummelo fruits (Supplementary Fig. S2), which is consistent with the expression level of *Cg4CL1*. Therefore, our research provides a better understanding of the regulation of multiple lignin biosynthetic genes by MYB58, suggesting that MYB58 is probably a TF marker responsible for granulation in citrus.

In addition to the high transcript level of *CgMYB58* in HR juice sacs, the other 23 TFs were significantly correlated with lignin content (Supplementary Table S2). Among these TFs, MYB46 and SND1 have been reported to regulate secondary cell wall-related processes^15,32^. In *Arabidopsis*, AtMYB46 has been reported to directly regulate AtMYB58 in lignin biosynthesis and AtSND1 is the upstream gene of AtMYB46^16,32^. Compared with *CgMYB46* and *CgSND1*, *CgMYB58* showed a higher expression level and a distinct expression pattern among HR and the other three pummelo cultivars at all developmental stages (Fig. 3c and Supplementary Table S2). Considering the RNA-seq results and previous research, CgMYB58 is thus the critical TF regulating lignin biosynthesis in pummelo. In addition, MYB58 and three other TFs, MYB4, MYB42, and WRKY12, were reported to affect secondary cell walls through the regulation of lignin in switchgrass^14^. Overall, to construct a regulatory network in the future, CgMYB58 can essentially be used as a bait to retrieve interacting regulators to help construct the hierarchical regulatory network and therefore comprehensively analyze the lignin accumulation leading to citrus granulation.

**Fig. 7:**
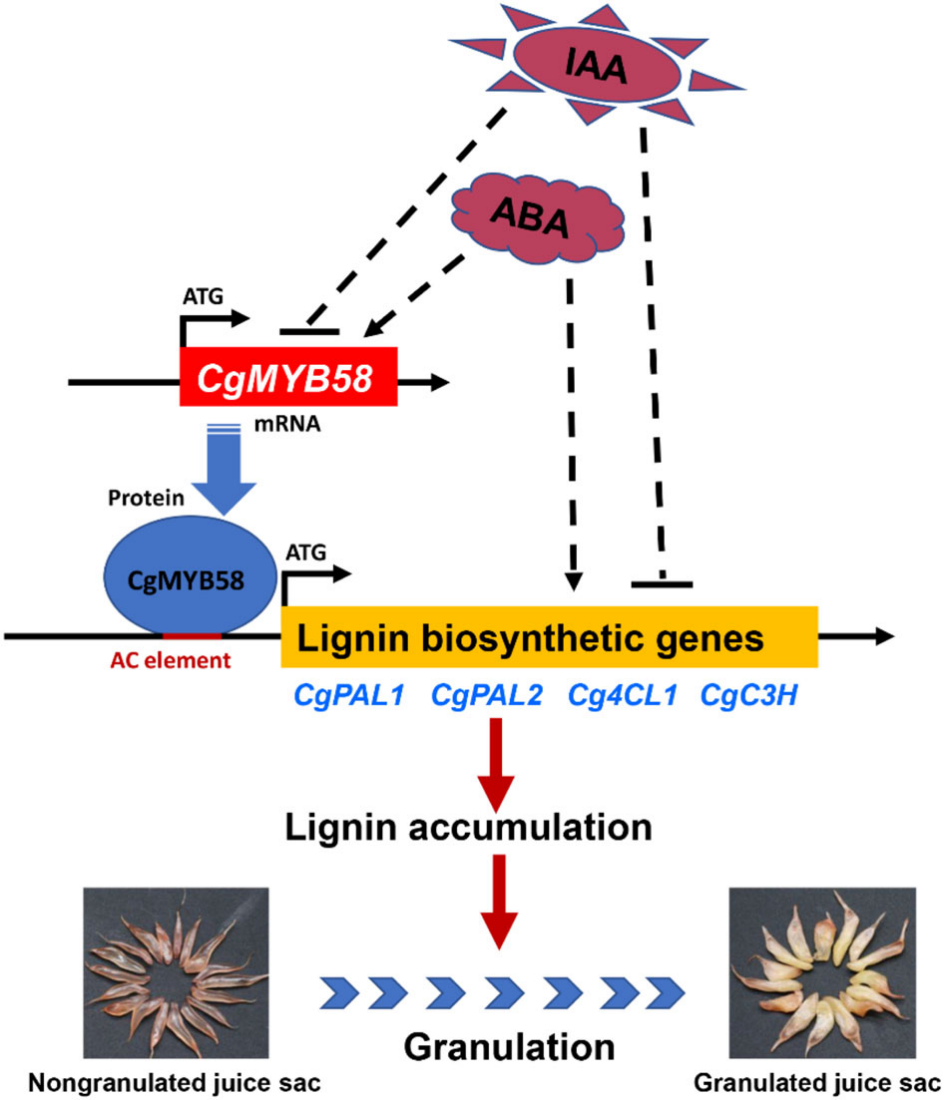
A proposed model for the regulation of lignin biosynthesis and granulation in citrus juice sacs by CgMYB58. In this model, CgMYB58 binds to AC elements in the promoter regions of four lignin biosynthetic genes (*CgPAL1*, *CgPAL2*, *Cg4CL1*, and *CgC3H*) and promotes their expression. ABA treatment increases the content of lignin by promoting the expression of *CgMYB58* and its direct target genes, while IAA treatment inhibits this process by repressing the expression of *CgMYB58* and its downstream genes

In addition, previous reports have revealed that some granulated citrus cultivars^9,10,28^ and Luoyangqing loquat^5,37^ accumulate high levels of both lignin and carotenoids. In our study, lignin showed high accumulation in granulated HR with the reddest flesh (Fig. 1), which also contained high levels of total and red carotenoids. This raised the question of whether specific carotenoids or their downstream metabolites are involved in the lignification process in HR. ABA is one of the downstream metabolites of carotenoids. Our previous reports showed that the ABA concentration was higher in pale-fleshed Feicui pummelo fruits than in red-fleshed Chuhong pummelo fruits at 150 DPA^25^. In addition, ABA has been reported to be involved in the regulation of lignin biosynthetic genes and TF regulators that respond to the lignin accumulation process in plants^34^. Accordingly, we found that juice sacs exhibited increases in lignin content and increases in the expression levels of *CgMYB58* and its target genes after being treated with ABA (Fig. 6b-d). Thus, ABA induced lignin biosynthesis by promoting the expression of *CgMYB58* and its target genes in HR, HB, and KP juice sacs (Fig. 7).

IAA has been shown to influence anthocyanin biosynthesis in apple^38^ and to disrupt the lignification process in the roots of *Asparagus officinalis*^33^. In this study, we first determined that IAA could significantly repress lignin biosynthesis in pummelo juice sacs, further inhibiting juice sac granulation (Fig. 7). Therefore, to avoid an unpleasant sensory texture, the application of exogenous IAA might downregulate the expression of *CgMYB58* and thus relieve granulation in the juice sacs, which provides a potential approach for regulating the granulation process by applying auxin-like plant growth regulators in the field.

## Materials and methods

### Plant materials

Fruits of HR pummelo, HB pummelo, FH pummelo, and KP pummelo were collected at 55, 85, 115, 145, 175, and 205 DPA from the National Citrus Breeding Center at Huazhong Agricultural University (Wuhan, China). In addition, fruits of nine pummelo cultivars at the commercially mature stage (~200 DPA) were also harvested (Supplementary Table S7). For each cultivar, 15–18 fruits were picked from at least three different healthy trees and divided randomly into three replicates. The juice sacs from each sample were immediately separated, frozen in liquid nitrogen, and then stored at −80 °C.

### Paraffin section observations of cell walls of the juice sacs

The reagents used for paraffin sectioning were analytically pure and purchased from Dingguo, Co. (China). The method was performed according to the methods of Li et al.^39^. The sections were stained with 1% phloroglucinol-hydrochloric acid or 0.1% toluidine blue solution for 3 min and then washed with 95% ethanol or deionized water for 10 min. The sections were subsequently examined and imaged using a Zeiss Axioscope A1 microscope (Swift Microscope World, California, USA) with a ×0.5 optical adapter. The images were captured and exported using ZEN 2.3 software (Zeiss).

### Lignin extraction and evaluation

According to the methods of Xu et al.^5^ (with minor modifications), the juice sacs (or calli) were ground into powder and 8 g (or 10 g for calli) of each sample was homogenized in 15 ml of washing buffer (100 mM K_2_HPO_4_/KH_2_PO_4_, 0.5% Triton X-100, 0.5% PVP-K30; pH 7.8). The mixture was washed on a shaker (GS-20, MiuLab, Hangzhou, China) at room temperature at 250 r.p.m./min for 30 min and then centrifuged (5000 × *g*, 25 °C) for 10 min (Avanti J-26 XP, Beckman Coulter, California, USA). Finally, the supernatant was discarded. Washing buffer was added three times, as described above. Next, the precipitates were washed with 20 ml of 100% methanol four times. The precipitates were subsequently dried at 60 °C in a vacuum rotary evaporator (5305FG924683, Eppendorf, Germany) overnight. Fifty milligrams (mg) of the lyophilized powder of the juice sac (for calli, 100 mg) was dissolved in 5.0 ml of 2.0 M HCl and 0.5 ml of thioglycolic acid. The mixture was then boiled within a water bath at 100 °C (LTT-600, Longyue, Shanghai, China) for 8 h, cooled on ice for 5 min, and centrifuged at 8000 × *g* for 20 min at 4 °C (H1850R, Hunan, China). The precipitates were washed with distilled water three times and dried thereafter at 60 °C in a vacuum rotary evaporator overnight. The sample was resuspended in 1.5 ml of 1.0 M NaOH and subsequently placed on a shaker at 100 r.p.m. at room temperature for 18 h. The solution was centrifuged at 10,000 × *g* for 20 min. Five hundred milliliters of the supernatant of was transferred to a new tube that contained 0.1 ml of concentrated HCl. The tubes were incubated at 4 °C for 4 h to precipitate the lignin thioglycolic acid, followed by centrifugation at 13,000 × *g* for 20 min at 4 °C (5404EP320017, Eppendorf, Germany). The precipitate was then dissolved in a 1 : 100 ml volume of 1.0 M NaOH. Absorbance was then measured at 280 nm using ultraviolet spectrophotometry (UV-1800, Japan), with 1.0 M NaOH used as a blank. The data were expressed on a fresh weight basis and three biological replicates were used for each sample.

### RNA isolation, library construction, and sequencing

The total RNA from the juice sacs was isolated according to the methods of Liu et al.^40^, while RNA from calli was isolated with TRIzol reagent (Aidlab Biotechnologies Co., Ltd, Beijing, China). The RNA from the HR, HB, KP, and FH fruit juice sacs at 55, 85, 115, 145, and 205 DPA was extracted, with three biological replicates included per sample. The following procedures were performed by Novogene Bioinformatics Technology, China. RNA integrity was assessed using a RNA Nano 6000 Assay Kit of a Bioanalyzer 2100 system (Agilent Technologies, CA, USA). A total amount of 3 µg of RNA per sample was used as input material for the RNA sample preparations. Sequencing libraries were generated using a NEBNext Ultra RNA Library Prep Kit for Illumina (NEB, USA) following the manufacturer’s recommendations and index codes were added to attribute sequences to each sample. The constructed RNA libraries were sequenced on an Illumina HiSeq 2500 platform in paired-end mode, with a read length of 150 bp.

### RNA-seq data analysis

Quality control of the raw sequencing data was performed with FastQC. Adapters were removed from reads and the data were cleaned based on phred scores using fastp. The clean data were mapped to the pummelo genome^31^. Read counts for each gene in each sample were extracted with the edgeR program. The FPKM of each gene was calculated based on its definition^41^. Differential expression analysis was performed using DESeq v1.18.0 in R v3.5.1^42^. We then used KOBAS software to test the statistical enrichment of DEGs in KEGG pathways. Gene Ontology (GO) enrichment analysis of DEGs was implemented by GOseq v1.38.0, in which gene length bias was corrected. GO terms with corrected *P*-values (*α* = 0.05) were considered significantly enriched for those DEGs^43^. For correlation analyses, Spearman’s rank correlations of the lignin content and FPKM of all the genes expressed in the KP, HB and HR pummelo juice sacs at the five developmental stages was calculated using the pspearman package. Genes with a correlation coefficient to lignin content ratio higher than 0.75 and a significant *P*-value (*α* = 0.05) were selected.

### cDNA synthesis and quantitative real-time PCR

A total of 0.5 μg of RNA was used for cDNA synthesis with a RevertAid First Strand cDNA Synthesis Kit (Thermo Fisher Scientific, USA). qRT-PCR was performed with a Roche LightCycler 480 system in conjunction with 23 LightCycler 480 SYBR Green Master Mix (Roche, USA), as described by Lu et al.^44^. The primers designed for qRT-PCR are listed in Supplementary Table S8 and are based on pummelo database gene sequences^31^. The actin gene was used as previously reported^26^. The qRT-PCR data were analyzed using the 2^-ΔCt^ analysis method.

### Gene cloning

The full-length coding DNA sequence (CDS) and 2.1 kb promoter region of *CgMYB58* and the promoter of lignin biosynthetic genes were amplified from the cDNA and DNA of various citrus cultivars. The sequences of the primers used are listed in Supplementary Table S8. Sequence results were obtained from ~20 and 10 clones amplified from cDNA and DNA templates, respectively.

### Phylogenetic analysis

The online version of ClustalW (https://www.genome.jp/tools-bin/clustalw) was utilized for multiple alignment of the amino acid sequences of CgMYB58 and the corresponding sequences of other species. Conserved domains were extracted using gblocks (http://www.phylogeny.fr/one_task.cgi?task_type=gblocks). MEGA 7.0 was subsequently used to construct a phylogenetic tree according to the maximum likelihood method. There were 1000 bootstrap replications and values higher than 50 for each node are presented in the tree.

### Subcellular localization analysis

The *CgMYB58* CDS without a stop codon was fused to green fluorescent protein (GFP) within a pM999 vector. Citrus (*C. limon* Burm “Eureka lemon”) mesophyll protoplasts were extracted carefully for transient transformation. Plasmids of *35S:CgMYB58-GFP* and *35S:OsGhd7-RFP* (nuclear marker) and pM999 empty vector plasmids were mixed together equally. These plasmid mixtures were then transferred into separate protoplasts. After 24 h, the florescence images were scanned via a confocal laser-scanning microscope (TCS SP2, Leica, Wetzlar, Germany).

### Transient transformation of *CgMYB58*

The *CgMYB58* overexpression construct (vector: pH7WG2D) and a pH7WG2D empty vector used as a control were introduced into *Agrobacterium tumefaciens* strain GV3101 separately. The GV3101 cells containing the constructs were suspended in infiltration buffer (50 mg of glucose, 1 ml of 50 mM MES, 1 ml of Na_3_PO_4_, and 1 µl of 1 M acetosyringone per 10 ml of buffer) at a concentration of OD_600_ = 0.8, and then injected into the mesocarp of KP and HR pummelo fruits. GFP signals were captured by an inversion fluorescence microscope (Olympus SZX7, Japan) equipped with a light source (H-150). Twelve fruits were transformed per treatment.

### Stable transformation of *CgMYB58* in citrus calli

The CDS of *CgMYB58* was isolated from HR and cloned into a pH7WG2D overexpression vector. The vector was then introduced into the *A. tumefaciens* strain EHA105 before it was transformed into calli of RM grapefruit (*C. paradisi*). The citrus callus transformation and growth conditions were performed according to the methods of Li et al.^45^, with minor modifications. Each transgenic callus was cultured in 500 ml of MT (Murashige and Tucker) media with vitamins (Coolaber Science & Technology, Beijing, China), supplemented with 80 μl of 50 mg ml^−1^ hygromycin B, whereas without hygromycin B was used for RM (the WT control). Each *CgMYB58* overexpression line and RM callus were subcultured six to seven times before analysis.

### Dual luciferase transcriptional activity assay

The dual LUC transcriptional activity assay procedure was modified from that of a previous study^44^. DNA sequences upstream of the translational start codon were amplified by PCR from genomic DNA of HR to generate 1062, 1101, 1298, and 939 bp promoter fragments for *CgPAL1*, *CgPAL2*, *Cg4CL1*, and *CgC3H*, respectively. The amplified fragments were subsequently inserted into a pGreenII 0800-LUC reporter vector, which was then introduced into *A. tumefaciens* GV3101 (pSoup-p19) competent cells. The effector vector was a *CgMYB58* overexpression (vector: pH7WG2D) construct, and a pH7WG2D empty vector was used as a control. The constructs were also introduced into GV3101 (pSoup-p19). The GV3101 cells containing effectors and reporters were mixed to a proportion of 4:1 and then injected into leaves of *Nicotiana benthamiana*. LUC activities were analyzed according to the methods of Lu et al.^44^ at 2.5 days after injection.

### Yeast one-hybrid assay analysis

Y1H assays were carried out using a Matchmaker Gold Yeast One-Hybrid system kit (Clontech, USA) and modified according to the methods of Lu et al.^44^. The promoter fragments of *CgPAL2* and *CgC3H* were cloned into a pAbAi vector to produce pAbAi-*CgPAL2*P and pAbAi-*CgC3H*P bait constructs, respectively. The bait plasmids were then linearized and integrated into Y1H Gold yeast, after which they were selected with synthetic dextrose media lacking uracil. The *CgMYB58* coding sequences were ligated into pGADT7 to generate an AD-CgMYB58 construct. pGADT7 empty vectors (AD-pGADT7), serving as negative controls, were then transferred separately into yeast cells containing bait constructs. The transformed yeast cells were diluted with 0, 400×, and 500× dilution series of aureobasidin A (AbA) and dotted on SD plates lacking leucine. The cells grew on both types of media containing prey proteins to allow the interaction of bait sequences.

### Electrophoretic mobility shift assays

EMSA assays were performed as described previously^26,46^. pMAL-c5x-CgMYB58 (with maltose binding protein tag) without a stop codon was expressed and purified as described by Lu et al.^44^. The 25–27 bp probes containing the AC elements in the promoters of *CgPAL1*, *CgPAL2*, *Cg4CL1*, and *CgC3H* were extracted and used as reference sequences to synthesize probes. 5’-FAM-labeled oligonucleotide probes were synthesized and labeled by Shanghai Sangon Company (Shanghai, China). The same oligonucleotides without labels were used as cold competitors. To perform binding reactions, the binding solution (0.1% NP-40, 1 mM benzamidine, 0.5 mM phenylmethylsulfonyl fluoride, 0.5 mM dithiothreitol, 50 μg ml^−1^ bovine serum albumin, 100 ng μl^−1^ poly(dI-dC)), 2 μl (0.5 mg ml^−1^) of purified maltose binding protein tag fused CgMYB58 and 1 μl of the 5’-FAM-labeled probe (10 μmol l^−1^) were mixed together and incubated at 4 °C for 45 min. For competition assays, the unlabeled oligonucleotides were incubated with protein and binding buffer at 4 °C for 45 min. Afterward, 1 μl of the 5’-FAM-labeled probe (10 μmol l^−1^) was added and incubated at 4 °C for 45 min. The samples were then loaded onto a prerun 6% polyacrylamide gel. Electrophoresis was performed at 4 °C using 0.5× Tris-borate-EDTA as an electrophoresis buffer in the dark for 1 h. Gel images were acquired using an Amersham Imager 600 (GE Healthcare, Tokyo, Japan).

### IAA and ABA treatments of pummelo juice sacs

The ABA- and auxin-responsive elements of gene promoters were predicted by the PlantCARE online database (http://bioinformatics.psb.ugent.be/webtools/plantcare/html/). The juice sacs of the KP, HB, and HR pummelo cultivars were collected at 145 DPA in 2019 and cultured in murashige and skoog media (4.43 g of murashige and skoog powder, 100 g of sucrose, and 7 g of agar per liter of medium), with IAA and ABA at a final concentration of 0.5 mM. After being cultured for 50 days on the media, the samples were collected for the evaluation of gene expression and lignin content. High-performance liquid chromatography-grade IAA and ABA standards were purchased from Coolaber Science & Technology (Beijing, China).

### Data analysis and software used

Metabolite and gene expression profiles were processed with Excel and GraphPad Prism 7. Heatmaps were processed by R with the gplot and pheatmap packages. Duncan’s multiple comparison test and Student’s *t*-test were conducted in conjunction with analysis of variance in SAS (SAS Institute, Inc., USA).

## Supplementary information

Supplementary Figures

Supplementary Table S1

Supplementary Table S2

Supplementary Table S3

Supplementary Table S6

Supplementary Table S4

Supplementary Table S8

Supplementary Table S5

Supplementary Table S7
