# Supplementary material for: The IAA- and ABA-responsive transcription factor CgMYB58 upregulates lignin biosynthesis and triggers juice sac granulation in pummelo
Source: Hortic Res. 2020 Sep 1;7:139. doi: 10.1038/s41438-020-00360-7 (PMC7458917; doi:10.1038/s41438-020-00360-7)
Supplement: Supplementary file 1 — Supplementary Figures [file 41438_2020_360_MOESM1_ESM.docx]

Supplementary figures for

**The IAA- and ABA-responsive transcription factor CgMYB58 upregulates lignin biosynthesis and triggers juice sac granulation in pummelo**

Meiyan Shi^1^, Xiao Liu^1^, Haipeng Zhang^1^, Zhenyu He^1^, Hongbin Yang^1^, Jiajing Chen^1^, Jia Feng^1^, Wenhui Yang^1^, Youwu Jiang^1^, Jia-Long Yao^2^, Cecilia Hong Deng^2^* and Juan Xu^1^*

Institution addresses:

^1^Key Laboratory of Horticultural Plant Biology, Ministry of Education, Wuhan, Hubei 430070, P.R. China.

^2^The New Zealand Institute for Plant and Food Research Limited, Private Bag 92169, Auckland 1142, New Zealand

*Corresponding authors: Cecilia Hong Deng ([cecilia.deng@plantandfood.co.nz](mailto:cecilia.deng@plantandfood.co.nz)) and Juan Xu (xujuan@mail.hzau.edu.cn)

Telephone number: 86-27-87286965; Fax number: 86-27-87280016.


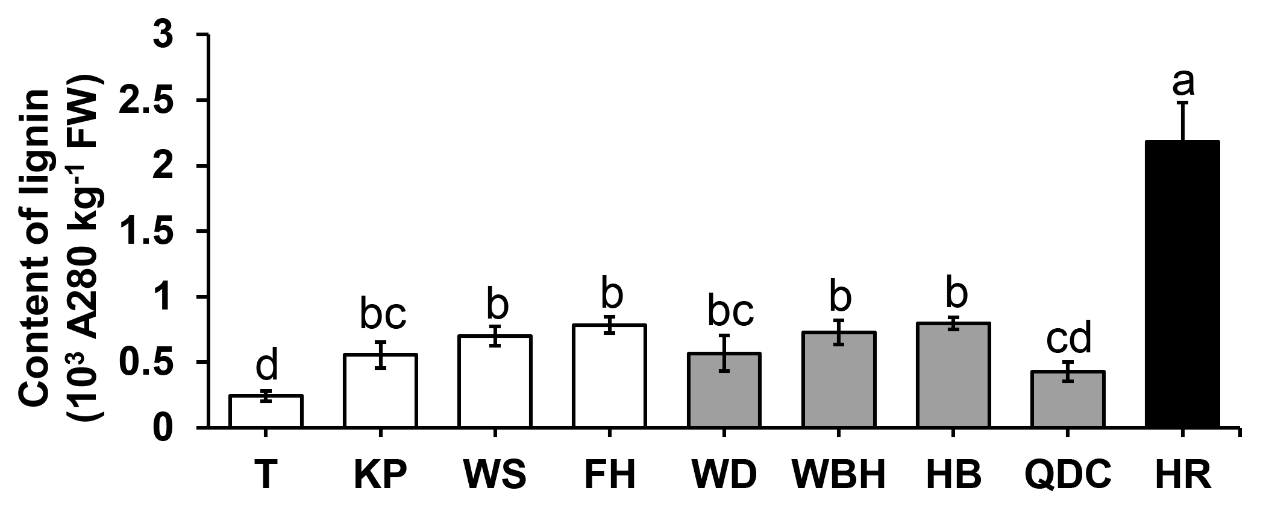


**Fig. S1** **The content of lignin in HR was greatly higher than other eight pummelo cultivars.** Lower-case letters above columns indicate significant differences among nine pummelo cultivars indicated by Duncan’s multiple comparisons (*P* < 0.05).


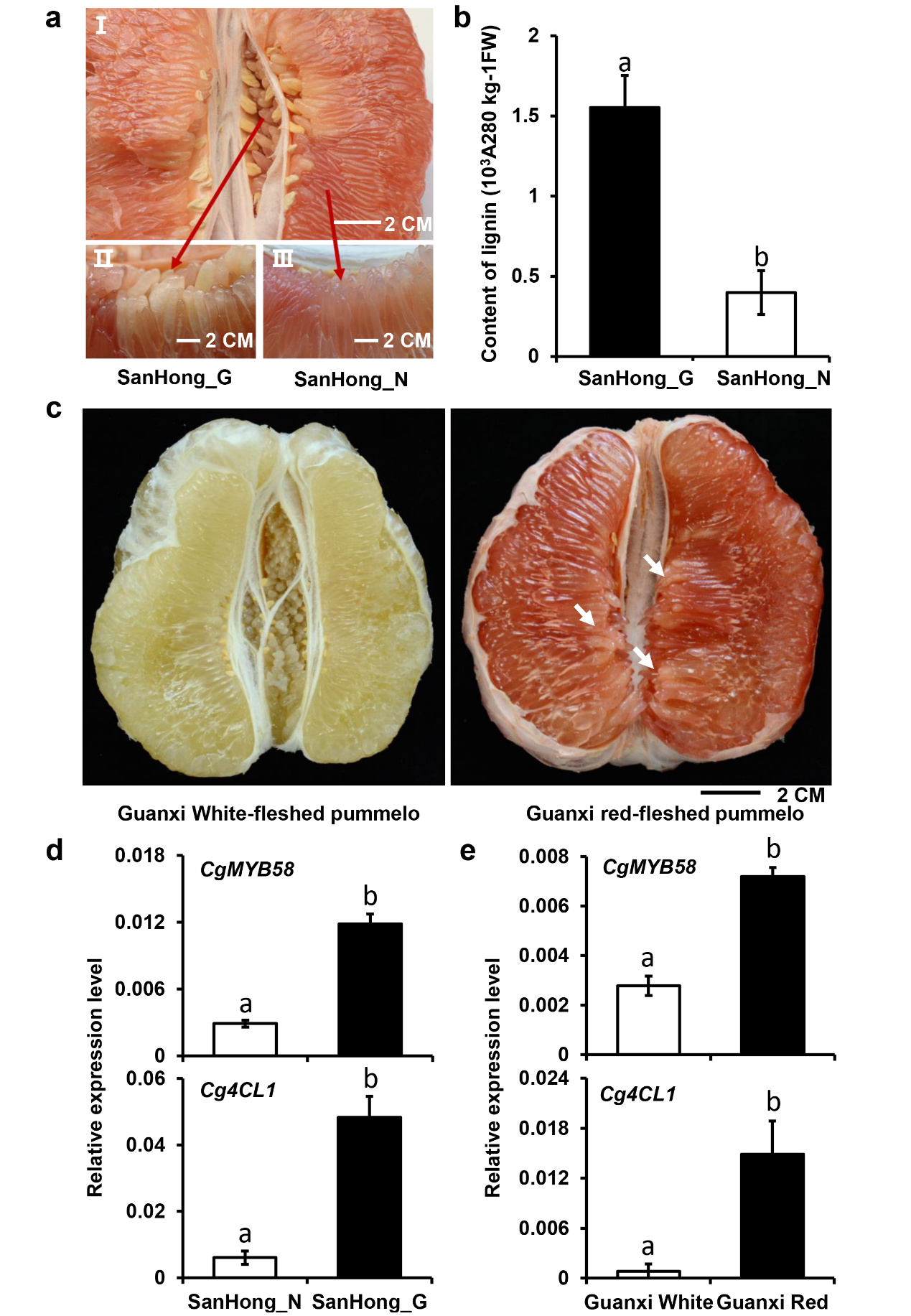


**Fig. S2** **Granulation phenotype and gene expression profiles in Guanxi pummelo (*C. grandis*).**

**a** Granulation phenotype of ‘SanHong’ Guanxi pummelo. ‘SanHong’ is a bud mutant of Guanxi pummelo (*C. grandis*)^9^ (I). SanHong_G: SanHong granulated juice sac (II), SanHong_N: SanHong normal juice sacs (III). **b** The lignin content was significantly higher in SanHong granulation juice sacs than in SanHong normal juice sacs. **c** Granulation phenotype in Guanxi pummelos (*C. grandis*). ‘Guanxi red-fleshed’ pummelo (right) was easier to be granulated (highlighted by white arrowhead) than ‘Guanxi white-fleshed’ pummelo (left), but with a less extent than SanHong Guanxi pummelo. **d** The expression level of *CgMYB58* was increased in SanHong granulated juice sacs and paralleled with that of *Cg4CL1* which is a critical gene in lignin biosynthetic pathway. **e** The expression levels of *CgMYB58* and *Cg4CL1* were dramatically higher in ‘Guanxi red-fleshed’ pummelo than in ‘Guanxi white-fleshed’ pummelo.


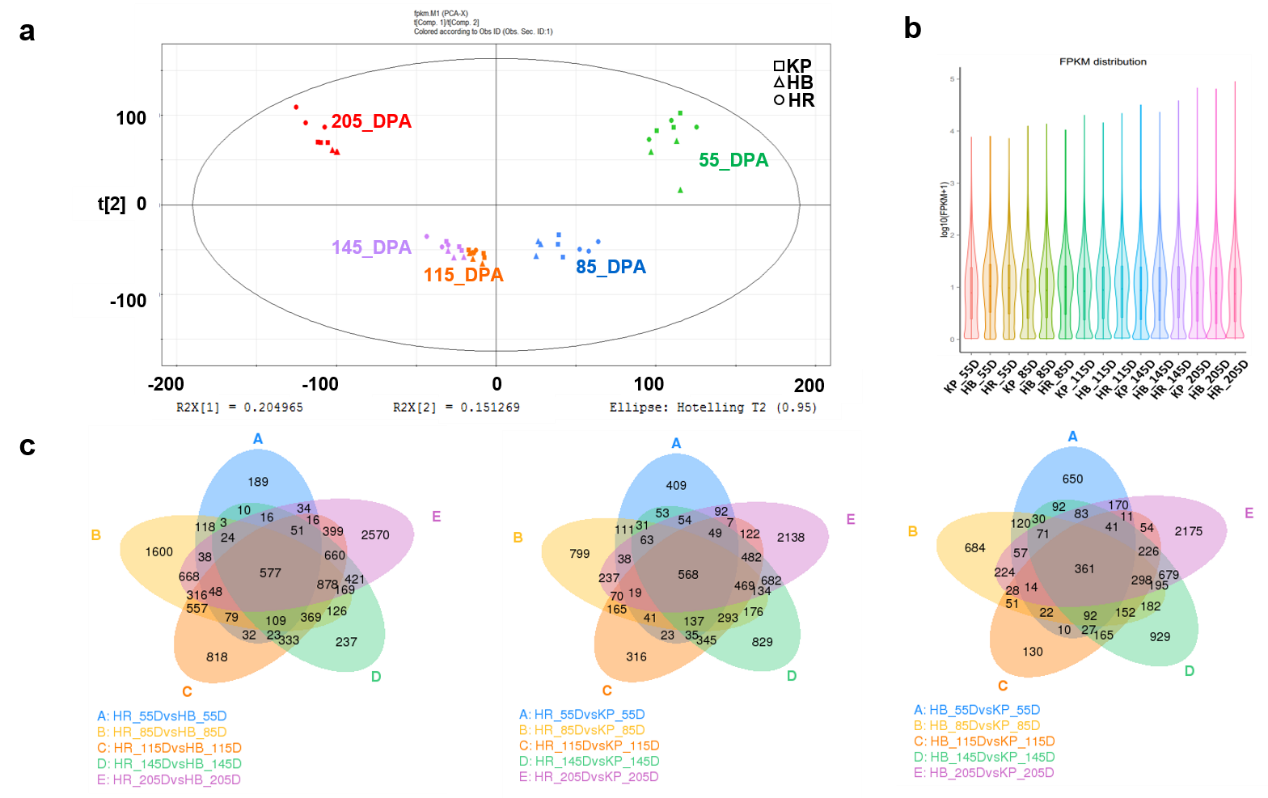


**Fig. S3 Global gene transcript profiles indicated pummelo genotypes at five developmental stages.**

**a** Principle component analysis (PCA) plot of the 45 samples dataset including three biological replicates. Different samples at the same stage were clustered together. **b** Distributions of Log10 (FPKM+1) in investigated samples. **c** Differentially expressed genes analysis among three pummelos at five developmental stages.


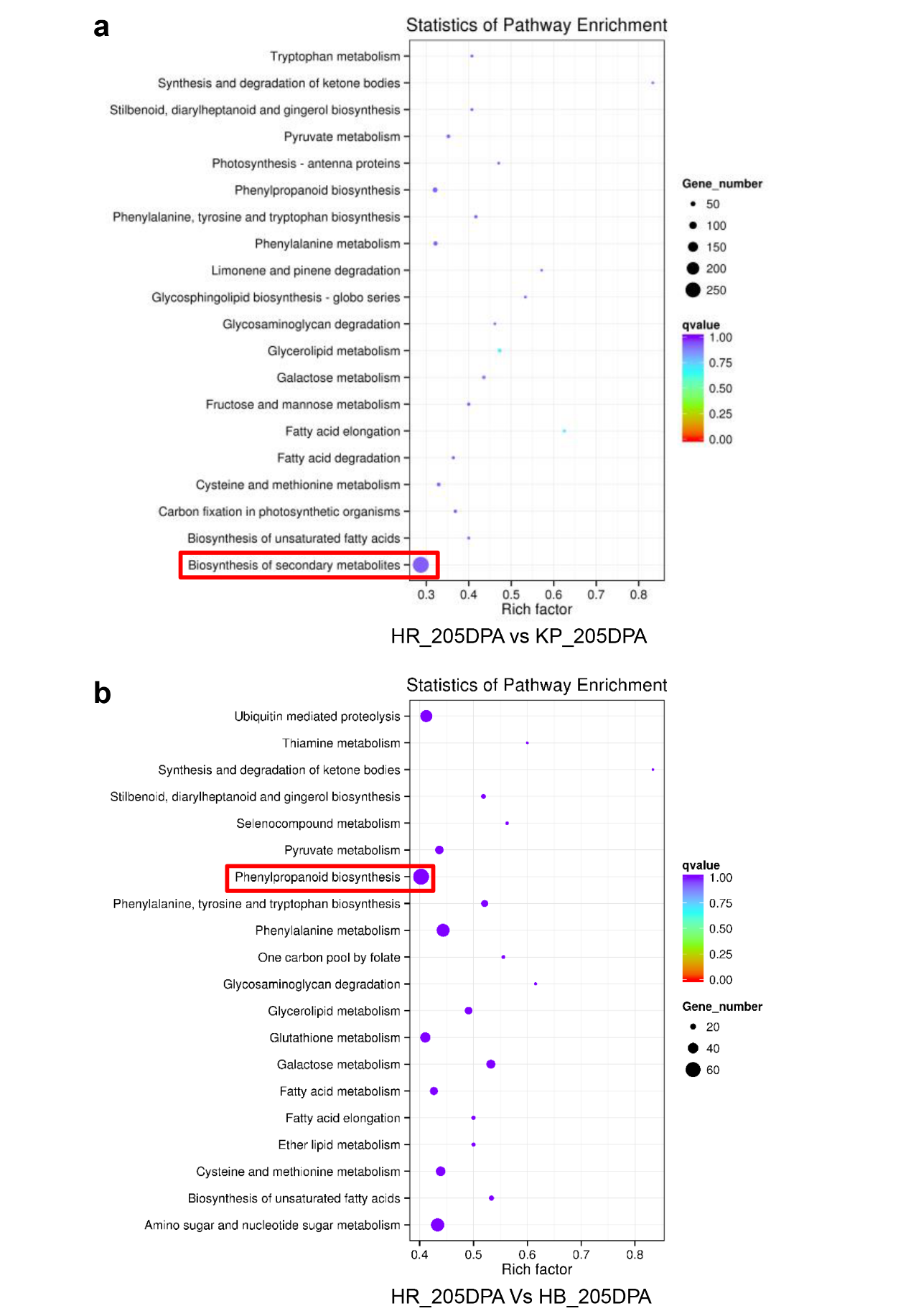


**Fig. S4** **KEGG annotation of differentially expressed genes (DEGs).**

**a** DEGs extracted from HR 205 DPA and KP 205 DPA. **b** DEGs extracted from HR 205 DPA and HB 205 DPA. DEGs were enriched in biosynthesis of secondary metabolites (a) and phenylpropanoid metabolites (b).


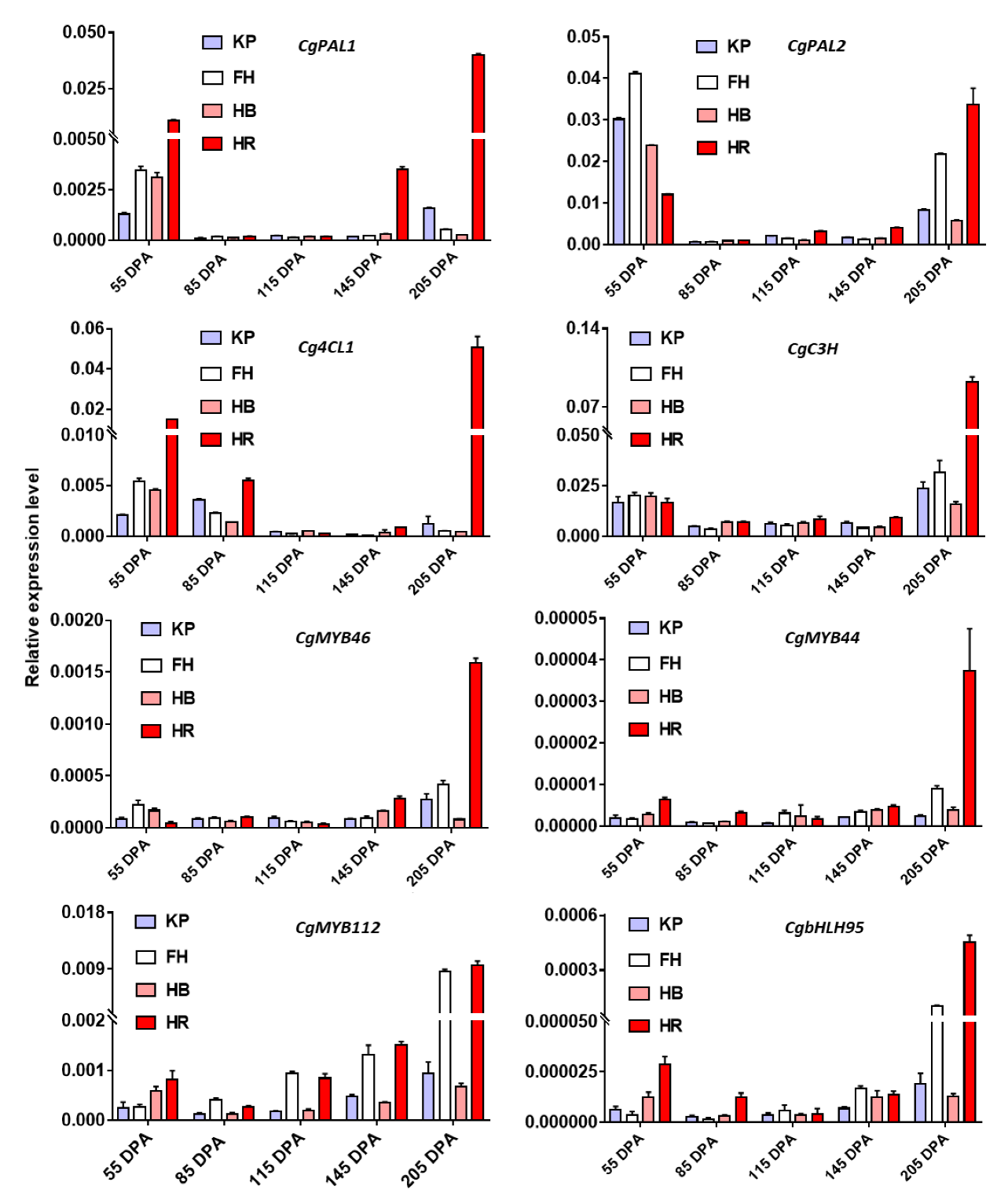


**Fig. S5** **Expression levels of genes associated with lignin biosynthesis indicated by qRT-PCR analysis**

Genes encoding four TFs and four lignin biosynthetic enzymes were selected for qRT-PCR analyses. qPCR results of the 8 genes were consistent with their RNA-seq results. The relative expression level was calculated with 2^-ΔCt^ analysis method. KP, ‘Kao Pan’; FH, ‘Fenghuang’, HB, ‘Hirado Buntan’; HR, ‘Huanong Red-fleshed’.


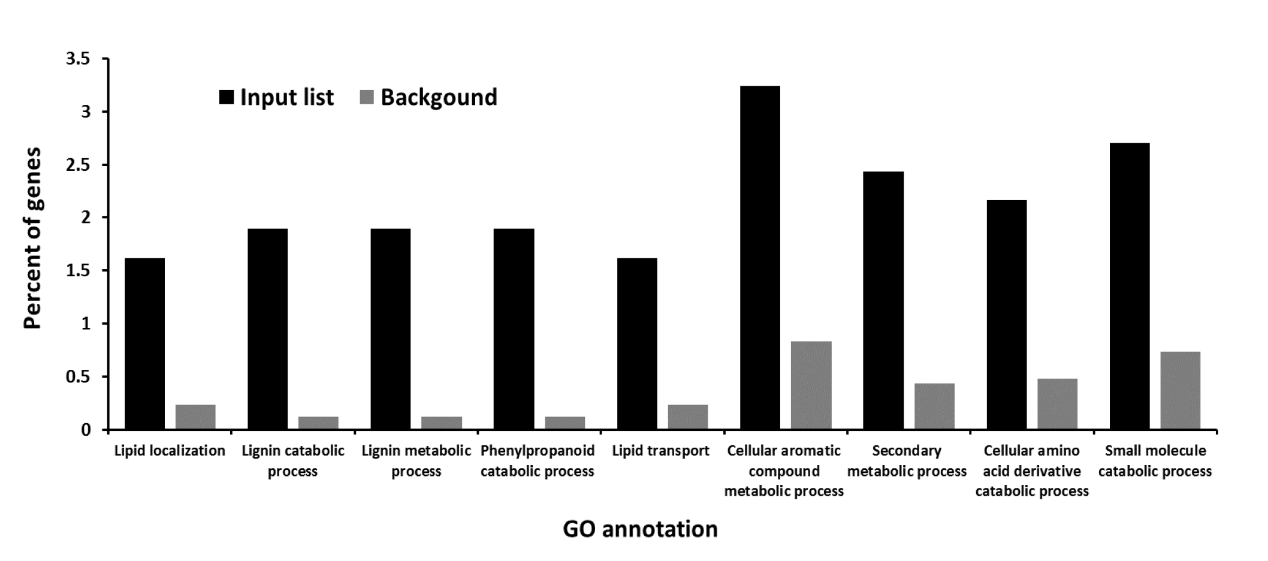


**Fig. S6 Genes correlating with lignin content variations as revealed in GO enrichment analysis.**

GO annotation of genes associated with lignin content variation in five developmental stages of HR, HB and KP. The results indicated that a great number of those genes are lignin catabolic and metabolic process related.


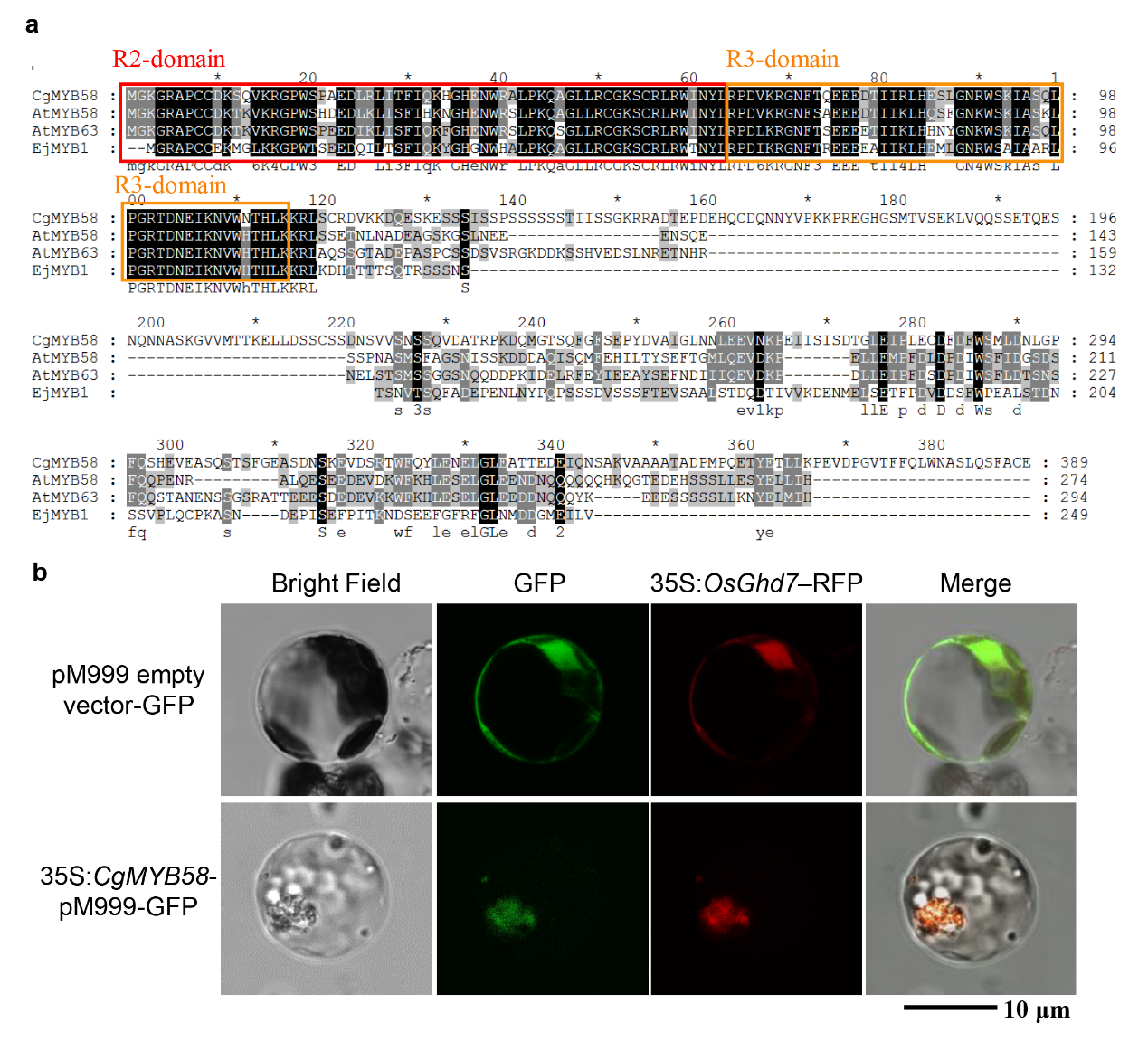


**Fig. S7** **Sequence analysis and subcellular localization of CgMYB58.**

**a** R2 and R3 domain were found in CgMYB58 coding sequence. **b** Subcellular localization analysis of CgMYB58 in citrus protoplasts. pM999 empty vector-GFP and 35S:*CgMYB58*-pM999-GFP were co-transformed with 35S: *OsGhd7*-CFP, respectively. OsGhd7 was used as a nuclear marker protein. CgMYB58-RFP, GFP signal; OsGhd7-RFP, RFP signal; Bright field, white light; Merged, combined GFP and RFP signals. Scale bars, 10 μm.

**
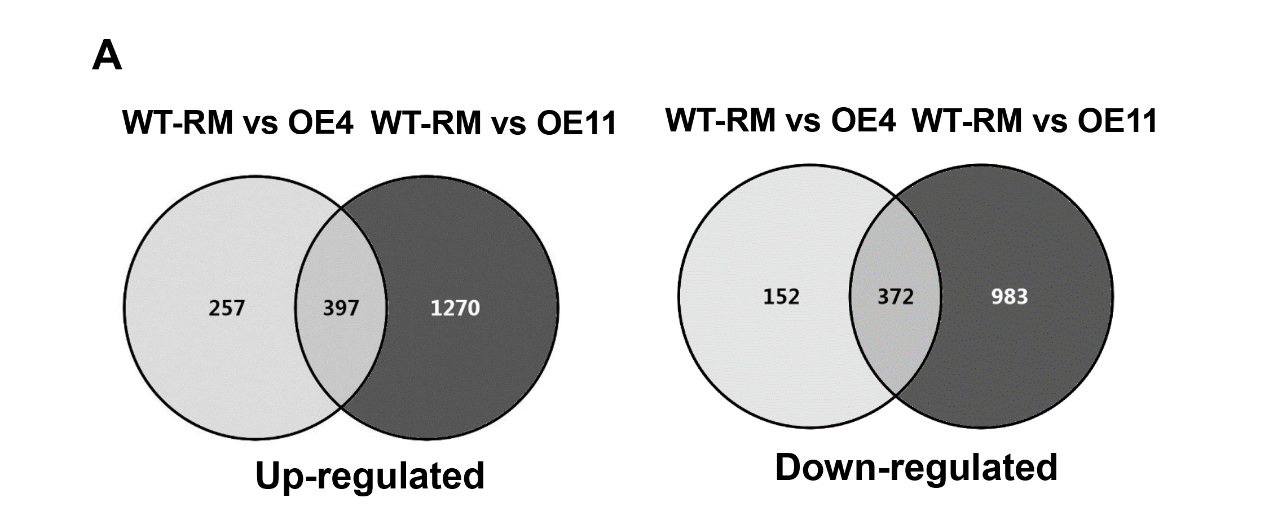
**

**Fig. S8 Venn diagram of RNA-Seq data in *CgMYB58*-OE citrus calli (OE4 & OE11) and their wild type (WT-RM).**

Venn diagrams of differentially expressed genes among WT-RM and two *CgMYB58*-OE lines. WT-RM vs OE4 share 397 up-regulated and 372 down-regulated genes with WT-RM vs OE11, respectively.


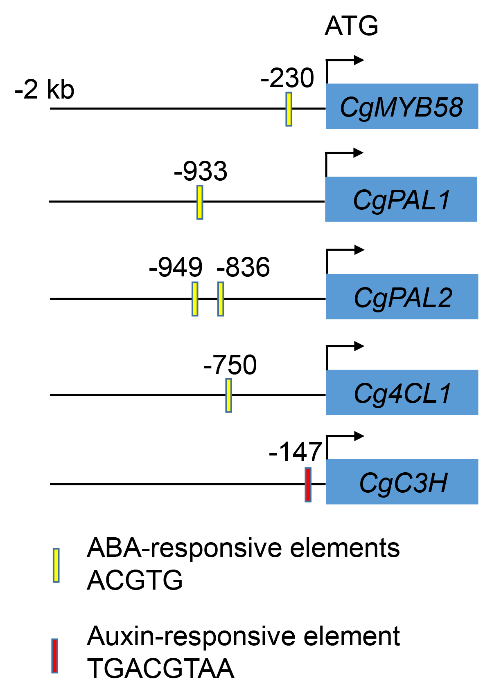


**Fig. S9 ABA- and auxin-responsive elements identified in the promoter of lignin related genes.**
