# Supplementary material for: The IAA- and ABA-responsive transcription factor CgMYB58 upregulates lignin biosynthesis and triggers juice sac granulation in pummelo
Source: Hortic Res. 2020 Sep 1;7:139. doi: 10.1038/s41438-020-00360-7 (PMC7458917; doi:10.1038/s41438-020-00360-7)
Supplement: Supplementary file 5 — Supplementary Table S6 [file 41438_2020_360_MOESM5_ESM.docx]

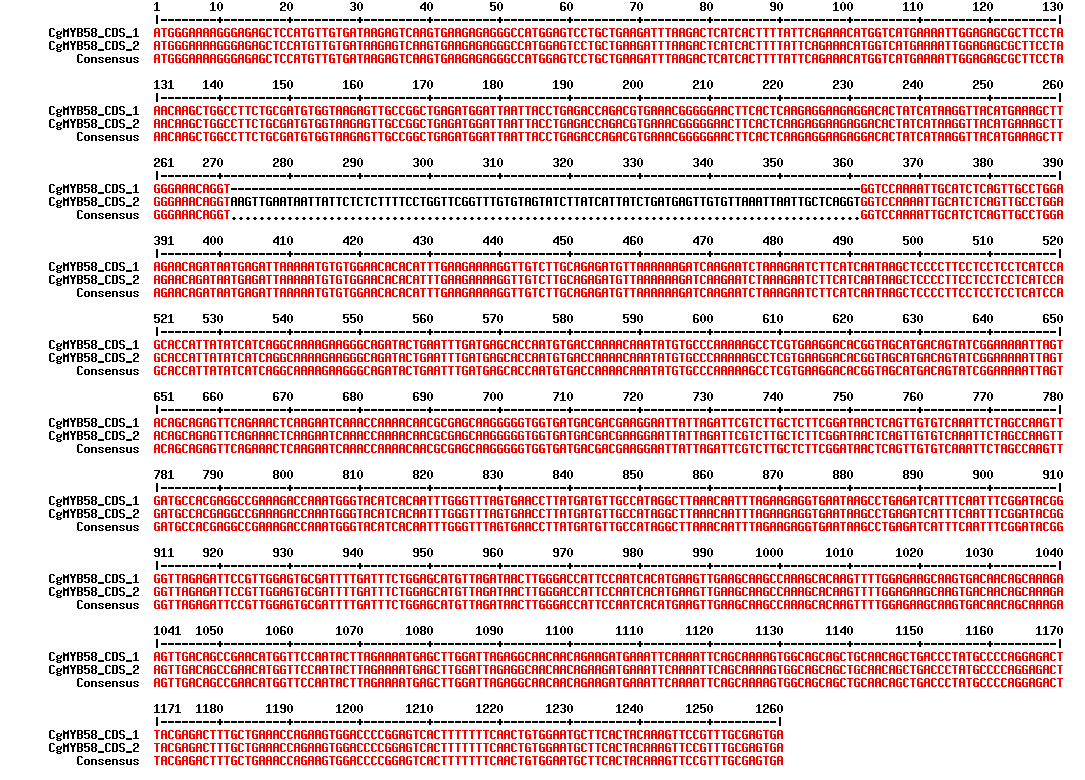
1. The Coding sequences of *CgMYB58*

>CgMYB58_CDS_1(***CgMYB58-1***)

ATGGGAAAAGGGAGAGCTCCATGTTGTGATAAGAGTCAAGTGAAGAGAGGGCCATGGAGTCCTGCTGAAGATTTAAGACTCATCACTTTTATTCAGAAACATGGTCATGAAAATTGGAGAGCGCTTCCTAAACAAGCTGGCCTTCTGCGATGTGGTAAGAGTTGCCGGCTGAGATGGATTAATTACCTGAGACCAGACGTGAAACGGGGGAACTTCACTCAAGAGGAAGAGGACACTATCATAAGGTTACATGAAAGCTTGGGAAACAGGTGGTCCAAAATTGCATCTCAGTTGCCTGGAAGAACAGATAATGAGATTAAAAATGTGTGGAACACACATTTGAAGAAAAGGTTGTCTTGCAGAGATGTTAAAAAAGATCAAGAATCTAAAGAATCTTCATCAATAAGCTcCCCTTCCTCCTCCTCATCCAGCACCATTATATCATCAGGCAAAAGAAGGGCAGATACTGAATTTGATGAGCACCAATGTGACCAAAACAAATATGTGCCCAAAAAGCCTCGTGAAGGACACGGTAGCATGACAGTATCGGAAAAATTAGTACAGCAGAGTTCAGAAACTCAAGAATCAAACCAAAACAACGCGAGCAAGGGGGTGGTGATGACGACGAAGGAATTATTAGATTCGTCTTGCTCTTCGGATAACTCAGTTGTGTCAAATTCTAGCCAAGTTGATGCCACGAGGCCGAAAGACCAAATGGGTACATCACAATTTGGGTTTAGTGAACCTTATGATGTTGCCATAGGCTTAAACAATTTAGAAGAGGTGAATAAGCCTGAGATCATTTCAATTTCGGATACGGGGTTAGAGATTCCGTTGGAGTGCGATTTTGATTTCTGGAGCATGTTAGATAACTTGGGACCATTCCAATCACATGAAGTTGAAGCAAGCCAAAGCACAAGTTTTGGAGAAGCAAGTGACAACAGCAAAGAAGTTGACAGCCGAACATGGTTCCAATACTTAGAAAATGAGCTTGGATTAGAGGCAACAACAGAAGATGAAATTCAAAATTCAGCAAAAGTGGCAGCAGCTGCAACAGCTGACCCTATGCCCCAGGAGACTTACGAGACTTTGCTGAAACCAGAAGTGGACCCCGGAGTCACTTTTTTTCAACTGTGGAATGCTTCACTACAAAGTTCCGTTTGCGAGTGA

> CgMYB58_CDS_2 (***CgMYB58-2***)

ATGGGAAAAGGGAGAGCTCCATGTTGTGATAAGAGTCAAGTGAAGAGAGGGCCATGGAGTCCTGCTGAAGATTTAAGACTCATCACTTTTATTCAGAAACATGGTCATGAAAATTGGAGAGCGCTTCCTAAACAAGCTGGCCTTCTGCGATGTGGTAAGAGTTGCCGGCTGAGATGGATTAATTACCTGAGACCAGACGTGAAACGGGGGAACTTCACTCAAGAGGAAGAGGACACTATCATAAGGTTACATGAAAGCTTGGGAAACAGGTAAGTTGAATAATTATTCTCTCTTTTCCTGGTTCGGTTTGTGTAGTATCTTATCATTATCTGATGAGTTGTGTTAAATTAATTGCTCAGGTGGTCCAAAATTGCATCTCAGTTGCCTGGAAGAACAGATAATGAGATTAAAAATGTGTGGAACACACATTTGAAGAAAAGGTTGTCTTGCAGAGATGTTAAAAAAGATCAAGAATCTAAAGAATCTTCATCAATAAGCTCCCCTTCCTCCTCCTCATCCAGCACCATTATATCATCAGGCAAAAGAAGGGCAGATACTGAATTTGATGAGCACCAATGTGACCAAAACAAATATGTGCCCAAAAAGCCTCGTGAAGGACACGGTAGCATGACAGTATCGGAAAAATTAGTACAGCAGAGTTCAGAAACTCAAGAATCAAACCAAAACAACGCGAGCAAGGGGGTGGTGATGACGACGAAGGAATTATTAGATTCGTCTTGCTCTTCGGATAACTCAGTTGTGTCAAATTCTAGCCAAGTTGATGCCACGAGGCCGAAAGACCAAATGGGTACATCACAATTTGGGTTTAGTGAACCTTATGATGTTGCCATAGGCTTAAACAATTTAGAAGAGGTGAATAAGCCTGAGATCATTTCAATTTCGGATACGGGGTTAGAGATTCCGTTGGAGTGCGATTTTGATTTCTGGAGCATGTTAGATAACTTGGGACCATTCCAATCACATGAAGTTGAAGCAAGCCAAAGCACAAGTTTTGGAGAAGCAAGTGACAACAGCAAAGAAGTTGACAGCCGAACATGGTTCCAATACTTAGAAAATGAGCTTGGATTAGAGGCAACAACAGAAGATGAAATTCAAAATTCAGCAAAAGTGGCAGCAGCTGCAACAGCTGACCCTATGCCCCAGGAGACTTACGAGACTTTGCTGAAACCAGAAGTGGACCCCGGAGTCACTTTTTTTCAACTGTGGAATGCTTCACTACAAAGTTCCGTTTGCGAGTGA

Supplementary table S6.1 Different *MYB58* transcripts in various pummelos (*Citrus grandis* Osbeck)

| **Cultivars** | **Abbreviation** | **Flesh color** | **Total Clones sequenced** | **Transcript /clones** |
| --- | --- | --- | --- | --- |
| ‘Chandler’ pummelo | QDC | Red | 22 | ***CgMYB58-1/22*** |
| ‘Huanong red-fleshed’ pummelo | HR | Red | 23 | ***CgMYB58-1/23*** |
| ‘Hirado Buntan’ pummelo | HB | Pink | 20 | ***CgMYB58-1/20*** |
| ‘WubuRed Fleshed’ pummelo | WBH | Red | 15 | ***CgMYB58-1/13, Cg****MYB58-2/2* |
| ‘Acidless’ pummelo | WS | Pale green | 19 | ***CgMYB58-1/19*** |
| ‘Fenghuang’ pummelo | FH | Pale green | 20 | ***CgMYB58-1/20*** |
| ‘Kao Pan’ pummelo | GB | Pale green | 20 | ***CgMYB58-1/20*** |
| ‘Thai’ pummelo | T | Pale yellow | 20 | ***CgMYB58-1/20*** |

2. Multiple alignments of *CgMYB58* promoters


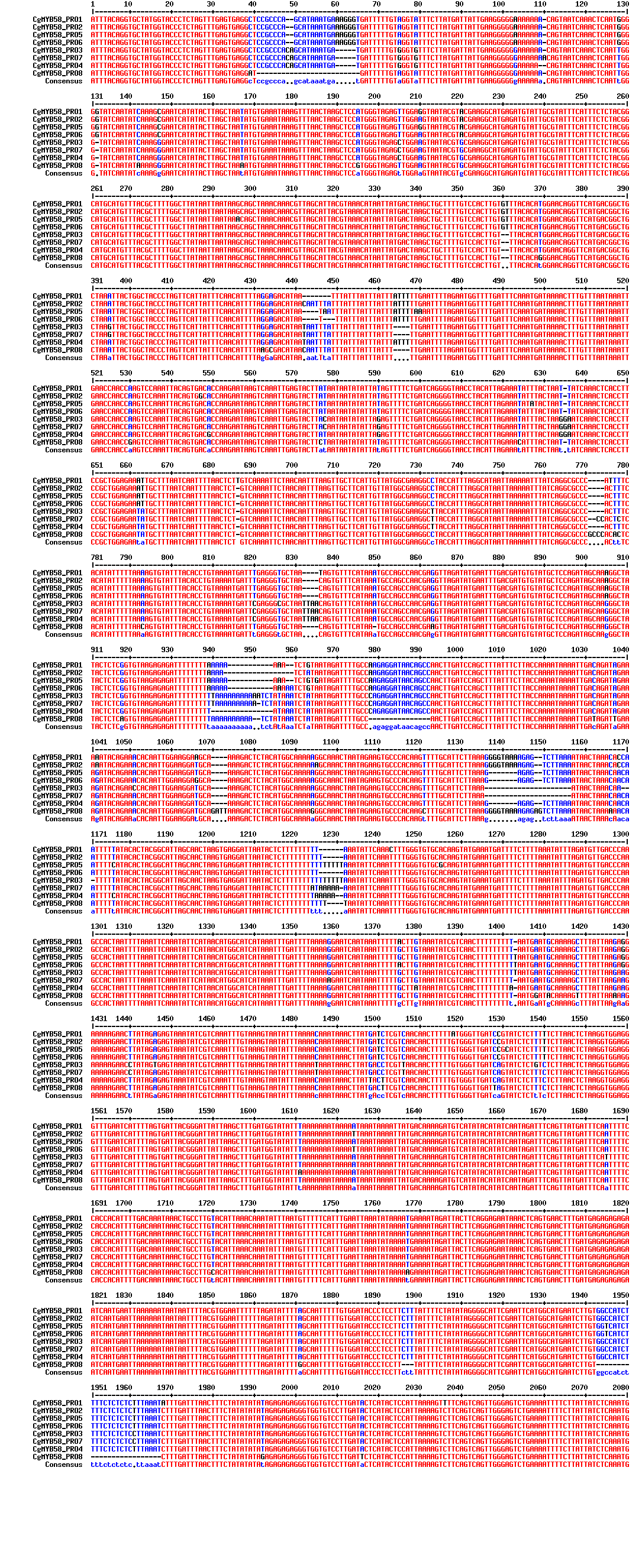

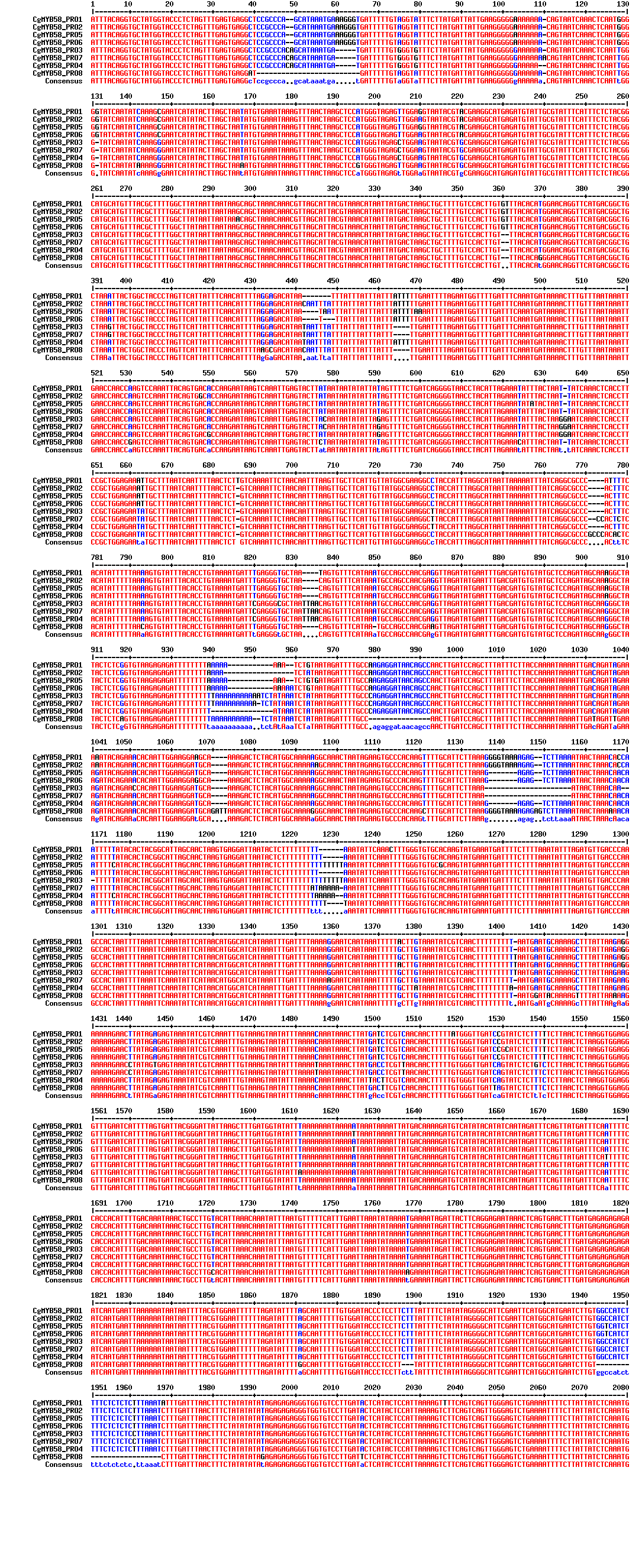

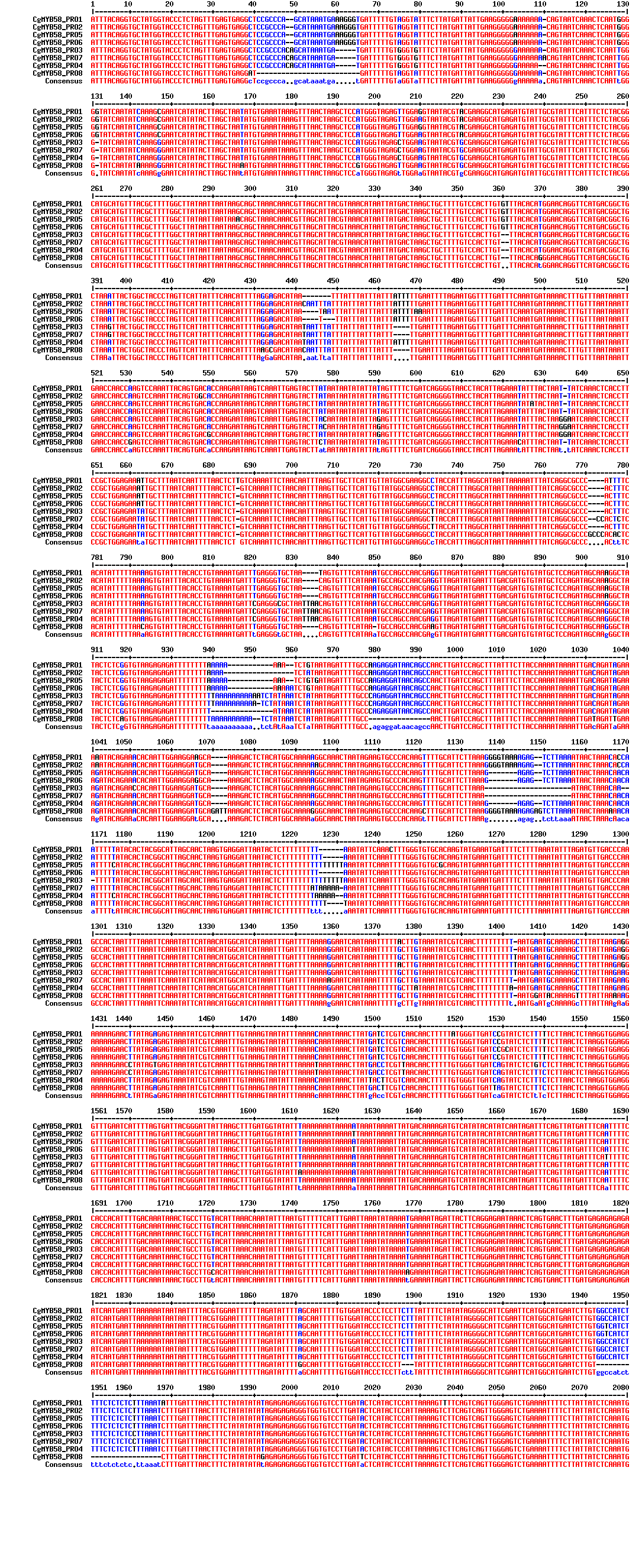

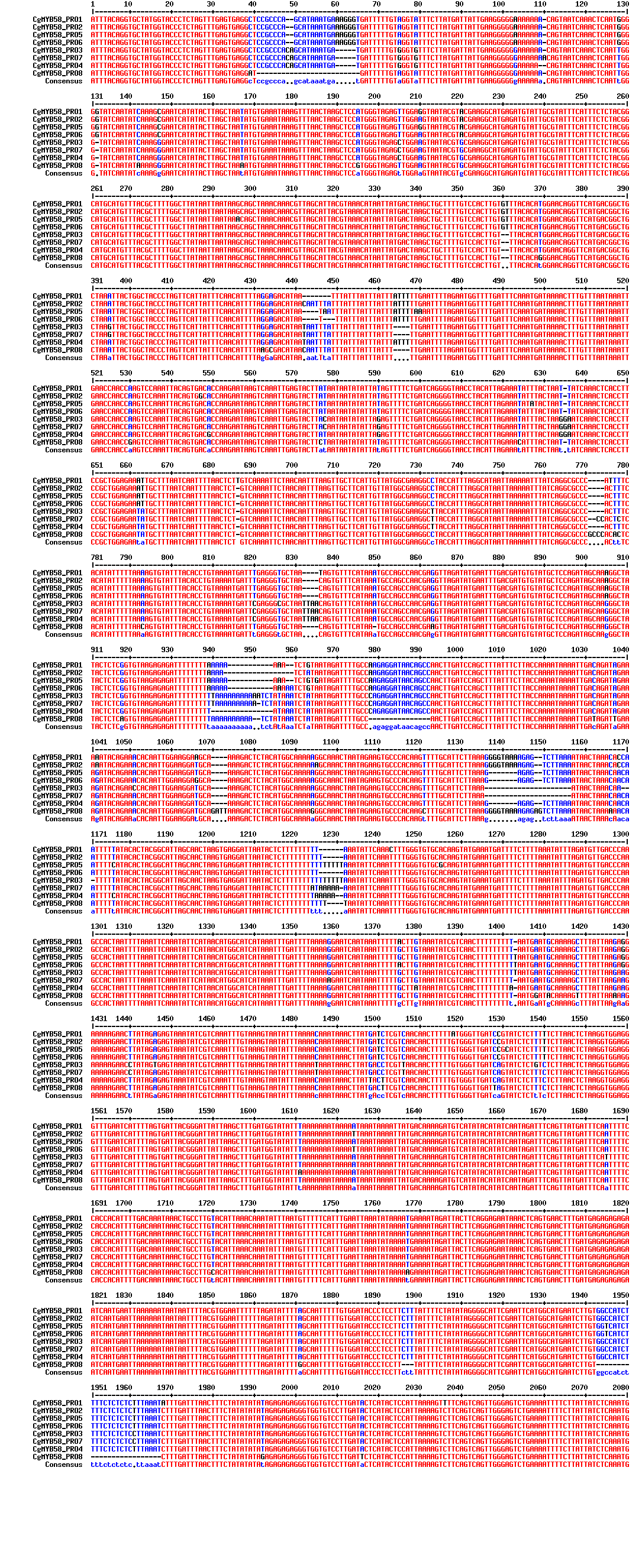


>MYB58_PRO1

ATTTACAGGTGCTATGGTACCCTCTAGTTTGAGTGAGGCTCCGCCCAGCATAAATGAAAGGGTGATTTTTGTAGGTATTTCTTATGATTATTGAAGGGGGAAAAAAACAGTAATCAAACTCAATGGGGGTATCAATATCAAAGCGAATCATATACTTAGCTAATATGTGAAATAAAGTTTAACTAAGCTCCATGGGTAGAGTTGGAGGTAATACGTACGAAGGCATGAGATGTATTGCGTATTTCATTTCTCTACGGCATGCATGTTTACGCTTTTGGCTTATAATTAATAAGCAGCTAAACAAACGTTAGCATTACGTAAACATAATTATGACTAAGCTGCTTTTGTCCACTTGTGTTTACACATGGAACAGGTTCATGACGGCTGCTAAATTACTGGCTACCCTAGTTCATTATTTCAACATTTTAGGAGACATAATTTATTTATTTATTTATTTTTGAATTTTAGAATGGTTTTGATTTCAAATGATAAAACTTTGTTTAATAAATTGAACCAACCAAGTCCAAATTACAGTGACACCAAGAATAAGTCAAATTGAGTACTTATAATAATATATTATAGTTTTCTGATCAGGGGTAACCTACATTAGAAATATTTACTAATTATCAAACTCACCTTCCGCTGGAGAAATTGCTTTAATCAATTTTAACTCTTGTCAAAATTCTAACAATTTAAGTTGCTTCATTGTTATGGCGAAGGCCTACCATTTAGGCATAATTAAAAATTTATCAGGCGCCCATTTTCACATATTTTTAAAAGTGTATTTACACCTGTAAAATGATTTGAGGGTGCTAATAGTGTTTCATAAATGCCAGCCAACGAGGTTAGATATGAATTTGACGATGTGTATGCTCCAGATAGCAAAGGCTATACTCTCGGTGTAAGAGAGATTTTTTTTAAAAAAAATCTGTAATAGATTTTGCCAAGAGGATAACAGCCAACTTGATCCAGCTTTATTTCTTACCAAAATAAAATTGACAGATAGAAAAATACAGAAACACAATTGGAAGGAAGCAAAAGACTCTACATGGCAAAAAGGCAAACTAATAGAAGTGCCCACAAGTTTTGCATTCTTAAAGGGGTAAAAGAGTCTTAAAATAACTAAACACCAATTTTTATACACTACGGCATTAGCAACTAAGTGAGGATTAATACTCTTTTTTTTTAAATATTCAAACTTTGGGTGTGCACAAGTATGAAATGATTTTCTTTTAAATATTTAGATGTTGACCCAAGCCACTAATTTTAAATTCAAATATTCATAACATGGCATCATAAATTTGATTTTAAAAGGAATCAATAAATTTTTACTTGTAAATATCGTCAACTTTTTTTTTAATGAATGCAAAAGCTTTATTAAGAGGAAAAAGAACTTATAGAGAGTAAATATCGTCAAATTTGTAAAGTAATATTTAAAACAAATAAACTTATGATCTCGTCAACAACTTTTTATGGGTTGATCCGTATCTCTTTTTCTTAACTCTAAGGTGGAGGGTTTGAATCATTTTAGTGATTACGGGATTATTAAGCTTTGATGGTATATTTAAAAAAATAAAAATAAATAAAATTATGACAAAAGATGTCATATACATATCAATAGATTTCAGTTATGATTTCAATTTTCCACCACATTTTGACAAATAAACTGCCTTGTACATTAAACAAATATTTAATGTTTTTCATTTGAATTAAATATAAAATGAAAATAGATTACTTCAGGAGAATAAACTCAGTGAACTTTGATGAGAGAGAGAATCAATGAATTAAAAAATAATAATTTTACGTGGAATTTTTTAGATATTTTAGCAATTTTTGTGGATACCCTCCTTCTTTATTTTCTATATAGGGGCATTCGAATTCATGGCATGAATCTTGTGGCCATCTTTTCTCTCTCTTTAAATATTTGATTTAACTTTCTATATATATAGAGAGAGGGTGGTGTCCTTGATACTCATACTCCATTAAAAGTTTTCAGTCAGTTGGGAGTCTGAAAATTTTCTTATTATCTCAAATG

>MYB58_PRO2

ATTTACAGGTGCTATGGTACCCTCTAGTTTGAGTGAGGCTCCGCCCAGCATAAATGAAAGGGTGATTTTTGTAGGTATTTCTTATGATTATTGAAGGGGGAAAAAAACAGTAATCAAACTCAATGGGGGTATCAATATCAAAGCGAATCATATACTTAGCTAATATGTGAAATAAAGTTTAACTAAGCTCCATGGGTAGAGTTGGAAGTAATACGTACGAAGGCATGAGATGTATTGCGTATTTCATTTCTCTACGGCATGCATGTTTACGCTTTTGGCTTATAATTAATAAGCAGCTAAACAAACGTTAGCATTACGTAAACATAATTATGACTAAGCTGCTTTTGTCCACTTGTGTTTACACATGGAACAGGTTCATGACGGCTGCTAAATTACTGGCTACCCTAGTTCATTATTTCAACATTTTAGGAGACATAACAATTTATTTATTTATTTATTTATTTTTGAATTTTAGAATGGTTTTGATTTCAAATGATAAAACTTTGTTTAATAAATTGAACCAACCAAGTCCAAATTACAGTGGCACCAAGAATAAGTCAAATTGAGTACTTATAATAATATATTATAGTTTTCTGATCAGGGGTAACCTACATTAGAAATATTTACTAATTATCAAACTCACCTTCCGCTGGAGAAATTGCTTTAATCAATTTTAACTCTGTCAAAATTCTAACAATTTAAGTTGCTTCATTGTTATGGCGAAGGCCTACCATTTAGGCATAATTAAAAATTTATCAGGCGCCCACTTTCACATATTTTTAAAAGTGTATTTACACCTGTAAAATGATTTGAGGGTGCTAACAGTGTTTCATAAATGCCAGCCAACGAGGTTAGATATGAATTTGACGATGTGTATGCTCCAGATAGCAAAGGCTATACTCTCGGTGTAAGAGAGATTTTTTTTAAAATCTATAATAGATTTTGCCAAGAGGATAACAGCCAACTTGATCCAGCTTTATTTCTTACCAAAATAAAATTGACAGATAGAAAAATACAGAAACACAATTGGAAGGATGCAAAAGACTCTACATGGCAAAAAAGCAAACTAATAGAAGTGCCCACAAGTTTTGCATTCTTAAAGGGGTAAAAGAGTCTTAAAATAACTAAACACCAATTTTTATACACTACGGCATTAGCAACTAAGTGAGGATTAATACTCTTTTTTTTTTAAATATTCAAATTTTGGGTGTGCACAAGTATGAAATGATTTTCTTTTAAATATTTAGATGTTGACCCAAGCCACTAATTTTAAATTCAAATATTCATAACATGGCATCATAAATTTGATTTTAAAAGGAATCAATAAATTTTTGCTTGTAAATATCGTCAACTTTTTTTTTAATGAATGCAAAAGCTTTATTAAGAGGAAAAAGAACTTATAGAGAGTAAATATCGTCAAATTTGTAAAGTAATATTTAAAACAAATAAACTTATGATCTCGTCAACAACTTTTTGTGGGTTGATCCGTATCTCTTTTTCTTAACTCTAAGGTGGAGGGTTTGAATCATTTTAGTGATTACGGGATTATTAAGCTTTGATGGTATATTTAAAAAAATAAAATTAAATAAAATTATGACAAAAGATGTCATATACATATCAATAGATTTCAGTTATGATTTCAATTTTCCACCACATTTTGACAAATAAACTGCCTTGTACATTAAACAAATATTTAATGTTTTTCATTTGAATTAAATATAAAATGAAAATAGATTACTTCAGGAGAATAAACTCAGTGAACTTTGATGAGAGAGAGAATCAATGAATTAAAAAATAATAATTTTACGTGGAATTTTTTAGATATTTTAGCAATTTTTGTGGATACCCTCCTTCTTTATTTTCTATATAGGGGCATTCGAATTCATGGCATGAATCTTGTGGCCATCTTTTCTCTCTCTTTAAATCTTTGATTTAACTTTCTATATATATAGAGAGAGGGTGGTGTCCTTGATACTCATACTCCATTAAAAGTCTTCAGTCAGTTGGGAGTCTGAAAATTTTCTTATTATCTCAAATG

>MYB58_PRO3

ATTTACAGGTGCTATGGTACCCTCTAGTTTGAGTGAGGCTCCGCCCACAGCATAAATGATGATTTTTGTGGGTGTTTCTTATGATTATTGAAGGGGGGAAAAAACAGTAATCAAACTCAATTGGGTATCAATATCAAAGGGAATCATATACTTAGCTAATATGTGAAATAAAGTTTAACTAAGCTCCATGGGTAGAGCTGGAAGTAATACGTGCGAAGGCATGAGATGTATTGCGTATTTCATTTCTCTACGGCATGCATGTTTACGCTTTTGGCTTATAATTAATAAGCAGCTAAACAAACGTTAGCATTACGTAAACATAATTATGACTAAGCTGCTTTTGTCCACTTGTTTACACATGGAACAGGTTCATGACGGCTGCTAAGTTACTGGCTACCCTAGTTCATTATTTCAACATTTTAGGAGACATAATAATTTATTTATTTATTTATTTTTGAATTTTAGAATGGTTTTGATTTCAAATGATAAAACTTTGTTTAATAAATTGAACCAACCAAGTCCAAATTACAGTGACACCAAGAATAAGTCAAATTGAGTACTTACAATAATATATTAGAGTTTTCTGATCAGGGGTAACCTACATTAGAAATATTTACTAAGGAATCAAACTCACCTTCCGCTGGAGAATATGCTTTAATCAATTTTAACTCTGTCAAAATTCTAACAATTTAAGTTGCTTCATTGTTATGGCGAAGGCTTACCATTTAGGCATAATTAAAAATTTATCAGGCGCCCACTTTCACATATTTTTAAAAGTGTATTTACACCTGTAAAATGATTCGAGGGCGCTAATTAACAGTGTTTCATAAATGCCAGCCAACGAGGTTAGATATGAATTTGACGATGTGTATGCTCCAGATAGCAAGGGCTATACTCTCGGTGTAAGAGAGATTTTTTTTTTAAAAAAAAAAATCTATAAATCTATAATAGATTTTGCCCAGAGGATAACAGCCAACTTGATCCAGCTTTATTTCTTACCAAAATAAAATTGACAGATAGAAAGATACAGAACCACAATTGGAAGGATGCAAAAGACTCTACATGGCAAAAAGGCAAACTAATAGAAGTGCCCACAAGTTTTGCATTCTTAAAATAACTAAACAATTTTTATACACTACGGCATTAGCAACTAAGTGAGGATTAATACTCTTTTTTTTTTTTTTTAAATATTCAAATTTTGGGTGTGCACAAGTATGAAATGATTTTCTTTTAAATATTTAGATGTTGACCCAAGCCACTAATTTTAAATTCAAATATTCATAACATGGCATCATAAATTTGATTTTAAAAGGAATCAATAAATTTTTGCTTGTAAATATCGTCAACTTTTTTTTTTAATGAATGCAAAAGCTTTATTAAGAAGAAAAAGAACCTATAGTGAGTAAATATCGTCAAATTTGTAAAGTAATATTTAAAATAAATAAACTTATGACCTCGTTAACAACTTTTTGTGGGTTGATCAGTATCTCTGTCTCTTAACTCTAAGGTGGAGGGTTTGAATCATTTTAGTGATTACGGGATTATTAAGCTTTGATGGTATATTTAAAAAAATAAAAATAAATAAAATTATGACAAAAGATGTCATATACATATCAATAGATTTCAGTTATGATTTCATTTTTCCACCACATTTTGACAAATAAACTGCCTTGTACATTAAACAAATATTTAATGTTTTTCATTTGAATTAAATATAAAATGAAAATAGATTACTTCAGGAGAATAAACTCAGTGAACTTTGATGAGAGAGAGAATCAATGAATTAAAAAATAATAATTTTACGTGGAATTTTTTAGATATTTTAGCAATTTTTGTGGATACCCTCCTTCTTTATTTTCTATATAGGGGCATTCGAATTCATGGCATGAATCTTGTGGCCATCTTTTCTCTCTCCTTAAATCTTTGATTTAACTTTCTATATATATAGAGAGAGGGTGGTGTCCTTGATACTCATACTCCATTAAAAGTCTTCAGTCAGTTGGGAGTCTGAAAATTTTCTTATTATCTCAAATG

>MYB58_PRO4

ATTTACAGGTGCTATGGTACCCTCTAGTTTGAGTGAGGCTCCGCCCACAGCATAAATGATGATTTTTGTGGGTGTTTCTTATGATTATTGAAGGGGGGAAAAACAGTAATCAAACTCAATTGGGTATCAATATCAAAGGGAATCATATACTTAGCTAATATGTGAAATAAAGTTTAACTAAGCTCCATGGGTAGAGCTGGAAGTAATACGTGCGAAGGCATGAGATGTATTGCGTATTTCATTTCTCTACGGCATGCATGTTTACGCTTTTGGCTTATAATTAATAAGCAGCTAAACAAACGTTAGCATTACGTAAACATAATTATGACTAAGCTGCTTTTGTCCACTTGTTTACACATGGAACAGGTTCATGACGGCTGCTAAATTACTGGCTACCCTAGTTCATTATTTCAACATTTTAGGAGACATAATAATTTATTTATTTATTTATTTATTTTTGAATTTTAGAATGGTTTTGATTTCAAATGATAAAACTTTGTTTAATAAATTGAACCAACCAAGTCCAAATTACAGTGACGCCAAGAATAAGTCAAATTGAGTACTTATAATAATATATTAGAGTTTTCTGATCAGGGGTAACCTACATTAGAAATATTTACTAAGGAATCAAACTCACCTTCCGCTGGAGAATATGCTTTAATCAATTTTAACTCTGTCAAAATTCTAACAATTTAAGTTGCTTCATTGTTATGGCGAAGGCTTACCATTTAGGCATAATTAAAAATTTATCAGGCGCCCACTTTCACATATTTTTAAAAGTGTATTTACACCTGTAAAATGATTCGAGGGTGCTAATTAACAGTGTTTCATAAATGCCAGCCAACGAGGTTAGATATGAATTTGACGATGTGTATGCTCCAGATAGCAAGGGCTATACTCTCGGTGTAAGAGAGATTTTTTTTTATAAATCTATAATAGATTTTGCCCAGAGGATAACAGCCAACTTGATCCAGCTTTATTTCTTACCAAAATAAAATTGACAGATAGAAAGATACAGAAACACAATTGGAAGGATGCAAAAGACTCTACATGGCAAAAAGGCAAACTAATAGAAGTGCCCACAAGTTTTGCATTCTTAAAGAGAGTCTTAAAATAACTAAACAACAATTTTCATACACTACGGCATTAGCAACTAAGTGAGGATTAATACTCTTTTTTTTAAAAAAAATATTCAAATTTTGGGTGTGCACAAGTATGAAATGATTTTCTTTTAAATATTTAGATGTTGACCCAAGCCACTAATTTTAAATTCAAATATTCATAACATGGCATCATAAATTTGATTTTAAAAGGAATCAATAAATTTTTGCTTATAAATATCGTCAACTTTTTTTTAAATGAATGCAAAAGCTTTATTAAGAAGAAAAAGAACTTATAGAGAGTAAATATCGTCAAATTTGTAAAGTAATATTTAAAACAAATAAACTTATTACTTCGTCAACAACTTTTTGTGGGTTGATCAGTATCTCTTTCTCTTAACTCTAAGGTGGAGGGTTTGAATCATTTTAGTGATTACGGGATTATTAAGCTTTGATGGTATATTAAAAAAAATAAAAATAAATAAAATTATGACAAAAGATGTCATATACATATCAATAGATTTCAGTTATGATTTCAATTTTCCACCACATTTTGACAAATAAACTGCCTTGTACATTAAACAAATATTTAATGTTTTTCATTTGAATTAAATATAAAATGAAAATAGATTACTTCAGGAGAATAAACTCAGTGAACTTTGATGAGAGAGAGAATCAATGAATTAAAAAATAATAATTTTACGTGGAATTTTTTAGATATTTTAGCAATTTTTGTGGATACCCTCCTTCTTTATTTTCTATATAGGGGCATTCGAATTCATGGCATGAATCTTGTGGCCATCTTTTCTCTCTCTTTAAATCTTTGATTTAACTTTCTATATATATAGAGAGAGGGTGGTGTCCTTGATACTCATACTCCATTAAAAGTCTTCAGTCAGTTGGGAGTCTGAAAATTTTCTTATTATCTCAAATG

>MYB58_PRO5

ATTTACAGGTGCTATGGTACCCTCTAGTTTGAGTGAGGCTCCGCCCAGCATAAATGAAAGGGTGATTTTTGTAGGTATTTCTTATGATTATTGAAGGGGGAAAAAAACAGTAATCAAACTCAATGGGGGTATCAATATCAAAGCGAATCATATACTTAGCTAATATGTGAAATAAAGTTTAACTAAGCTCCATGGGTAGAGTTGGAGGTAATACGTACGAAGGCATGAGATGTATTGCGTATTTCATTTCTCTACGGCATGCATGTTTACGCTTTTGGCTTATAATTAATAAACAGCTAAACAAACGTTAGCATTACGTAAACATAATTATGACTAAGCTGCTTTTGTCCACTTGTGTTTACACATGGAACAGGTTCATGACGGCTGCTAAATTACTGGCTACCCTAGTTCATTATTTCAACATTTTAGGAGACATAATAATTTATTTATTTATTTATTTTAAAATTTTAGAATGGTTTTGATTTCAAATGATAAAACTTTGTTTAATAAATTGAACCAACCAAGTCCAAATTACAGTGACACCAAGAATAAGTCAAATTGAGTACTTATAATAATATATTATAGTTTTCTGATCAGGGGTAACCTACATTAGAAATATATACTAATTATCAAACTCACCTTCCGCTGGAGAAATTGCTTTAATCAATTTTAACTCTGTCAAAATTCTAACAATTTAAGTTGCTTCATTGTTATGGCGAAGGCCTACCATTTAGGCATAATTAAAAATTTATCAGGCGCCCACTTTCACATATTTTTAAAAGTGTATTTACACCTGTAAAATGATTTGAGGGTGCTAACAGTGTTTCATAAATGCCAGCCAACGAGGTTAGATATGAATTTGACGATGTGTATGCTCCAGATAGCAAAGGCTATACTCTCGGTGTAAGAGAGATTTTTTTTAAAAAAAATCTGTGATAGATTTTGCCAAGAGGATAACAGCCAACTTGATCCAGCTTTATTTCTTACCAAAATAAAATTGACAGATAGAAAGATACAGAAACACAATTGGAAGGATGCAAAAGACTCTACATGGCAAAAAGGCAAACTAATAGAAGTGCCCACAAGTTTTGCATTCTTAAAGAGAGTCTTAAAATAACTAAACAACAATTTTCATACACTACGGCATTAGCAACTAAGTGAGGATTAATACTCTTTTTTTTTTTTTTTAAATATTCAAATTTTGGGTGTGCGCAAGTATGAAATGATTTTCTTTTAAATATTTAGATGTTGACCCAAGCCACTAATTTTAAATTCAAATATTCATAACATGGCATCATAAATTTGATTTTAAAAGGAATCAATAAATTTTTGCTTGTAAATATCGTCAACTTTTTTTTTTAATGAATGCAAAAGCTTTATTAAGAGGAAAAAGAACTTATAGAGAGTAAATATCGTCAAATTTGTAAAGTAATATTTAAAACAAATAAACTTATGATCTCGTCAACAACTTTTTGTGGGTTGATCCGCATCTCTTTTTCTTAACTCTAAGGTGGAGGGTTTGAATCATTTTAGTGATTACGGGATTATTAAGCTTTGATGGTATATTTAAAAAAATAAAAATAAATAAAATTATGACAAAAGATGTCATATACATATCAATAGATTTCAGTTATGATTTCAATTTTCCACCACATTTTGACAAATAAACTGCCTTGTACATTAAACAAATATTTAATGTTTTTCATTTGAATTAAATATAAAATGAAAATAGATTACTTCAGGAGAATAAACTCAGTGAACTTTGATGAGAGAGAGAATCAATGAATTAAAAAATAATAATTTTACGTGGAATTTTTTAGATATTTTAGCAATTTTTGTGGATACCCTCCTTCTTTATTTTCTATATAGGGGCATTCGAATTCATGGCATGAATCTTGTGGTCATCTTTTCTCTCTCTTTAAATCTTTGATTTAACTTTCTATATATATAGAGAGAGGGTGGTGTCCTTGATACTCATACTCCATTAAAAGTCTTCAGTCAGTTGGGAGTCTGAAAATTTTCTTATTATCTCAAATG

>MYB58_PRO6

ATTTACAGGTGCTATGGTACCCTCTAGTTTGAGTGAGGCTCCGCCCAGCATAAATGAAAGGGTGATTTTTGTAGGTATTTCTTATGATTATTGAAGGGGGAAAAAAACAGTAATCAAACTCAATGGGGGTATCAATATCAAAGCGAATCATATACTTAGCTAATATGTGAAATAAAGTTTAACTAAGCTCCATGGGTAGAGTTGGAAGTAATACGTACGAAGGCATGAGATGTATTGCGTATTTCATTTCTCTACGGCATGCATGTTTACGCTTTTGGCTTATAATTAATAAGCAGCTAAACAAACGTTAGCATTACGTAAACATAATTATGACTAAGCTGCTTTTGTCCACTTGTGTTTACACATGGAACAGGTTCATGACGGCTGCTAAATTACTGGCTACCCTAGTTCATTATTTCAACATTTTAGGAGACATAATTTATTTATTTATTTATTTTTGAATTTTAGAATGGTTTTGATTTCAAATGATAAAACTTTGTTTAATAAATTGAACCAACCAAGTCCAAATTACAGTGACACCAAGAATAAGTCAAATTGAGTACTTATAATAATATATTATAGTTTTCTGATCAGGGGTAACCTACATTAGAAATATTTACTAATTATCAAACTCACCTTCCGCTGGAGAAATTGCTTTAATCAATTTTAACTCTGTCAAAATTCTAACAATTTAAGTTGCTTCATTGTTATGGCGAAGGCCTACCATTTAGGCATAATTAAAAATTTATCAGGCGCCCACTTTCACATATTTTTAAAAGTGTATTTACACCTGTAAAATGATTTGAGGGTGCTAACAGTGTTTCATAAATGCCAGCCAACGAGGTTAGATATGAATTTGACGATGTGTATGCTCCAGATAGCAAAGGCTATACTCTCGGTGTAAGAGAGATTTTTTTTAAAAAAAAAATCTGTAATAGATTTTGCCAAGAGGATAACAGCCAACTTGATCCAGCTTTATTTCTTACCAAAATAAAATTGACAGATAGAAAGATACAGAAACACAATTGGAAGGAGGCAAAAGACTCTACATGGCAAAAAGGCAAACTAATAGAAGTGCCCACAAGTTTTGCATTCTTAAAGAGAGTCTTAAAATAACTAAACAACAATTTTTATACACTACGGCATTAGCAACTAAGTGAGGATTAATACTCTTTTTTTTTAAATATTCAAATTTTGGGTGTGCACAAGTATGAAATGATTTTCTTTTAAATATTTAGATGTTGACCCAAGCCACTAATTTTAAATTCAAATATTCATAACATGGCATCATAAATTTGATTTTAAAAGGAATCAATAAATTTTTACTTGTAAATATCGTCAACTTTTTTTTTTAATGAATGCAAAAGCTTTATTAAGAGGAAAAAGAACTTATAGAGAGTAAATATCGTCAAATTTGTAAAGTAATATTTAAAACAAATAAACTTATGATCTCGTCAACAACTTTTTGTGGGTTGATCCGTATCTCTTTTTCTTAACTCTAAGGTGGAGGGTTTGAATCATTTTAGTGATTACGGGATTATTAAGCTTTGATGGTATATTTAAAAAAATAAAATTAAATAAAATTATGACAAAAGATGTCATATACATATCAATAGATTTCAGTTATGATTTCAATTTTCCACCACATTTTGACAAATAAACTGCCTTGTACATTAAACAAATATTTAATGTTTTTCATTTGAATTAAATATAAAATGAAAATAGATTACTTCAGGAGAATAAACTCAGTGAACTTTGATGAGAGAGAGAATCAATGAATTAAAAAATAATAATTTTACGTGGAATTTTTTAGATATTTTAGCAATTTTTGTGGATACCCTCCTTCTTTATTTTCTATATAGGGGCATTCGAATTCATGGCATGAATCTTGTGGTCATCTTTTCTCTCTCTTTAAATCTTTGATTTAACTTTCTATATATATAGAGAGAGGGTGGTGTCCTTGATACTCATACTCCATTAAAAGTCTTCAGTCAGTTGGGAGTCTGAAAATTTTCTTATTATCTCAAATG

>MYB58_PRO7

ATTTACAGGTGCTATGGTACCCTCTAGTTTGAGTGAGGCTCCGCCCACAGCATAAATGATGATTTTTGTGGGTGTTTCTTATGATTATTGAAGGGGGGAAAAAAACAGTAATCAAACTCAATTGGGTATCAATATCAAAGGGAATCATATACTTAGCTAATATGTGAAATAAAGTTTAACTAAGCTCCATGGGTAGAGCTGGAAGTAATACGTGCGAAGGCATGAGATGTATTGCGTATTTCATTTCTCTACGGCATGCATGTTTACGCTTTTGGCTTATAATTAATAAGCAGCTAAACAAACGTTAGCATTACGTAAACATAATTATGACTAAGCTGCTTTTGTCCACTTGTTTACACATGGAACAGGTTCATGACGGCTGCTAAGTTACTGGCTACCCTAGTTCATTATTTCAACATTTTAGGAGACATAATAATTTATTTATTTATTTATTTTTGAATTTTAGAATGGTTTTGATTTCAAATGATAAAACTTTGTTTAATAAATTGAACCAACCAAGTCCAAATTACAGTGACACCAAGAATAAGTCAAATTGAGTACTTACAATAATATATTAGAGTTTTCTGATCAGGGGTAACCTACATTAGAAATATTTACTAAGGAATCAAACTCACCTTCCGCTGGAGAATATGCTTTAATCAATTTTAACTCTGTCAAAATTCTAACAATTTAAGTTGCTTCATTGTTATGGCGAAGGCTTACCATTTAGGCATAATTAAAAATTTATCAGGCGCCCCCACTCTCACATATTTTTAAAAGTGTATTTACACCTGTAAAATGATTCGAGGGTGCTAATTAACAGTGTTTCATAAATGCCAGCCAACGAGGTTAGATATGAATTTGACGATGTGTATGCTCCAGATAGCAAGGGCTATACTCTCGGTGTAAGAGAGATTTTTTTTTTAAAAAAAAAATCTATAAATCTATAATAGATTTTGCCCAGAGGATAACAGCCAACTTGATCCAGCTTTATTTCTTACCAAAATAAAATTGACAGATAGAAAGATACAGAAACACAATTGGAAGGATGCAAAAGACTCTACATGGCAAAAAGGCAAACTAATAGAAGTGCCCACAAGTTTTGCATTCTTAAAATAACTAAACAACAATTTTTATACACTACGGCATTAGCAACTAAGTGAGGATTAATACTCTTTTTTTATAAAAAAAATATTCAAATTTTGGGTGTGCACAAGTATGAAATGATTTTCTTTTAAATATTTAGATGTTGACCCAAGCCACTAATTTTAAATTCAAATATTCATAACATGGCATCATAAATTTGATTTTAAAAAGAATCAATAAATTTTTGCTTGTAAATATCGTCAACTTTTTTTTTAATGAATGCAAAAGCTTTATTAAGAAGAAAAAGAACCTATAGAGAGTAAATATCGTCAAATTTGTAAAGTAATATTTAAAATAAATAAACTTATGACCTCGTTAACAACTTTTTGTGGGTTGATCAGTATCTCTTTCTCTTAACTCTAAGGTGGAGGGTTTGAATCATTTTAGTGATTACGGGATTATTAAGCTTTGATGGTATATTTAAAAAAATAAAAATAAATAAAATTATGACAAAAGATGTCATATACATATCAATAGATTTCAGTTATGATTTCAATTTTCCACCACATTTTGACAAATAAACTGCCTTGTACATTAAACAAATATTTAATGTTTTTCATTTGAATTAAATATAAAATGAAAATAGATTACTTCAGGAGAATAAACTCAGTGAACTTTGATGAGAGAGAGAATCAATGAATTAAAAAATAATAATTTTACGTGGAATTTTTTAGATATTTTAGCAATTTTTGTGGATACCCTCCTTCTTTATTTTCTATATAGGGGCATTCGAATTCATGGCATGAATCTTGTGGCCATCTTTTCTCTCTCCTTAAATCTTTGATTTAACTTTCTATATATATAGAGAGAGGGTGGTGTCCTTGATACTCATACTCCATTAAAAGTCTTCAGTCAGTTGGGAGTCTGAAAATTTTCTTATTATCTCAAATG

>MYB58_PRO8

ATTTACAGGTGCTATGGTACCCTCTAGTTTGAGTGAGGATGATTTTTGTAGGTATTTCTTATGATTATTGAAGGGGGGAAAAAACAGTAATCAAACTCAATTGGGTATCAATATAAAAGGGAATCATATACTTAGCTAAAATGTGAAATAAAGTTTAACTAAGCTCCGTGGGTAGAGTTGGAAGTAATACGTGCGAAGGCATGAGATGTATTGCGTATTTCATTTCTCTACGGCATGCATGTTTACGCTTTTGGCTTATAATTAATAAGCAGCTAAACAAACGTTAGCATTACGTAAACATAATTATGACTAAGCTGCTTTTGTCCACTTGTTTACACAGGGAACAGGTTCATGACGGCTGCTAAATTACTGGCTACCCTAGTTCATTATTTCAACATTTTAAGCGACATAACAATTTATTTATTTATTTATTTTTGAATTTTAGAATGGTTTTGATTTCAAATGATAAAACTTTGTTTAATAAATTGAACCAACCGAGTCCAAATTACAGTGACACCAAGAATAAGTCAAATTGAGTACTTCTAATAATATATTATAGTTTTCTGATCAGGGGTAACCTACATTAGAAACATTTACTAATTATCAAACTCACCTTCCGCTGGAGAATATGCTTTAATCAATTTTAACTCTGTCAAAATTCTAACAATTTAAGTTGCTTCATTGTTATGGCGAAGGCCTACCATTTAGGCATAATTAAAAATTTATCAGGCGCCCGCCCACACTCACATATTTTTAACAGTGTATTTACACCTGTAAAATGATTTGAGGGTGCTAACAGTGTTTCATAATGCCAGCCAACGAAGTTAGATATGAATTTGACGATGTGTATGCTCCAGATAGCAAGGGCTATACTCTCAGTGTAAGAGAGATTTTTTTTTAAAAAAAAAATCTATAAATCTATAATAGATTTTGCCAACTTGATCCAGCTTTATTTCTTACCAAAATAAAATTGATAGATTGAAAGATACAGAAACACAATTGGAAGGATGCAGATTAAAGACTCTACATGGCAAAAGGGCAAACTAATAGAAGTGCCCACAAGCTTTGCATTCTTAAAGGGGTAAAAGAGAGTCTTAAAATAACTAAAAAACAATTTTTATACACTACGGCATTAGCAACTAAGTGAGGATTAATACTCTTTTTTTTTTTTAATATTCAAATTTTGGGTGTGCACAAGTATGAAATGATTTTCTTTTAAATATTTAGATGTTGACCCAAGCCACTAATTTTAAATTCAAATATTCATAACATGGCATCATAAATTTGATTTTAAAAGGAATCAATAAATTTTTGCTTGTAAATATCGTCAACTTTTTTTTTAATGGATACAAAAGTTTTATTAAAAAGAAAAAGAACTTATAGAGAGTAAATATCGTCAAATTTGTAAAGTAATATTTAAAACAAATAAACTTATGACTTCGTCAACAACTTTTTGTGGGTTGATTAGTATCTCTTTCTCTTAACTCTAAGGTGGAGGGTTTGAATCATTTTAGTGATTACGGGATTATTAAGCTTTGATGGTATATTTAAAAAAATAAAAATAAATAAAATTATGACAAAAGATGTCATATACATATCAATAGATTTCAGTTATGATTTCAATTTTCCACCACATTTTGACAAATAAACTGCCTTGCACATTAAACAAATATTTAATGTTTTTCATTTGAATTAAATATAAAAAGAAAATAGATTACTTCAGGAGAATAAACTCAGTGAACTTTGATGAGAGAGAGAATCAATGAATTAAAAAATAATAATTTTACGTGGAATTTTTTAGATATTTTGGCAATTTTTGTGGATACCCTCCTTTATTTTCTATATAGGGGCATTCGAATTCATGGCATGAATCTTGTCTTTGATTTAACTTTCTATATATAGAGAGAGAGGGTGGTGTCCTTGATTCTCATACTCCATTAAAAGTCTTCAGTCAGTTGGGAGTCTGAAAATTTTCTTATTATCTCAAATG

Note: ABA response elements were highlighted with purple, and the start codon were highlighted with blue.

Supplementary table S6.2 Different *MYB58* promoters in various citrus

| **Cultivars** | Scientific name | **Total Clones sequenced** | **Transcript /clones** |
| --- | --- | --- | --- |
| ‘Chandler’ pummelo | *C. grandis* Osbeck. | 11 | ***MYB58-PRO1/11*** |
| ‘Huanong red-fleshed’ pummelo | *C. grandis* Osbeck. | 11 | ***MYB58-PRO2/6, MYB58-PRO1/5*** |
| ‘Hirado Buntan’ pummelo | *C. grandis* Osbeck. | 12 | ***MYB58-PRO2/12*** |
| ‘Wanbai’ pummelo | *C. grandis* Osbeck. | 12 | ***MYB58-PRO2/12*** |
| ‘Thai’ pummelo | *C. grandis* Osbeck. | 14 | ***MYB58-PRO5/6, MYB58-PRO6/8*** |
| ‘Fenghuang’ pummelo | *C. grandis* Osbeck. | 12 | ***MYB58-PRO2/6, MYB58-PRO5/6*** |
| ‘Feicui’ pummelo | *C. grandis* Osbeck. | 9 | ***MYB58-PRO2/5, MYB58-PRO5/4*** |
| ‘Shatian’ pummelo | *C. grandis* Osbeck. | 11 | ***MYB58-PRO2/6, MYB58-PRO5/5*** |
| ‘Lime’ | *C. qurantifolia Swingle* | 11 | ***MYB58-PRO7/7, MYB58-PRO8/4*** |
| ‘Tarocco Blood’ orange | *C. sinensis* Osbeck. | 11 | ***MYB58-PRO3/3, MYB58-PRO7/8*** |
| ‘Washington Navel’ orange | *C. sinensis* Osbeck. | 12 | ***MYB58-PRO3/4, MYB58-PRO7/8*** |
| ‘Guoqing No 1’ satsuma mandarin | *C. reticulata* Blanco | 6 | ***MYB58-PRO3/1, MYB58-PRO4/5*** |
