# Supplementary material for: The IAA- and ABA-responsive transcription factor CgMYB58 upregulates lignin biosynthesis and triggers juice sac granulation in pummelo
Source: Hortic Res. 2020 Sep 1;7:139. doi: 10.1038/s41438-020-00360-7 (PMC7458917; doi:10.1038/s41438-020-00360-7)
Supplement: Supplementary file 7 — Supplementary Table S8 [file 41438_2020_360_MOESM7_ESM.docx]

| Gene name | Gene_ID | The position of MYB58 specific AC elements in the promoter region before ATG |
| --- | --- | --- |
| *PAL1（1062bp）* | Cg6g001770 | -383，-248，-211，-207 |
| *PAL2 (1489bp)* | Cg6g001740 | -419 |
| *PAL3 (2000bp)* | Cg8g019990 | -214 |
| *PAL4 (2000bp)* | Cg8g020000 | -231 |
| *PAL5 (2000bp)* | Cg7g006780 | -356 |
| *4CL1 (1298bp)* | Cg2g026340 | -224 |
| *C3H (939bp)* | Cg6g017470 | -1966, -1954, -1415, -457，-405，-15 |
| *CCOAOMT1 (2000bp)* | Cg1g005540 | -1780 |
| *CCOAOMT2 (2000bp)* | Cg6g002800 | -219 |
| *CCOAOMT4 (2000bp)* | Cg8g004310 | -550，-152 |

Supplementary table S8 Loci of MYB58 specific AC elements in the promoter region (bp upstream of the ATG) of lignin biosynthetic genes

>CgPAL1_Pro

GGAAAGAAAAACGAAACGCCAAAATAATTGAAGCAAATTTATAAAAATATATTTGAACTTTGGAGTGGGTCCTACTAAAGACAAAAGAGTAGCGTCATCTAAGACAACTTTTTGAGTCCTACAACTGGCACGTGCCAATCGTCTTATAACAACTGACATAATTAAATAAATACATTTAAAAAAATTAAATAAATAGGTTGATTTAAAGGAATTTTAATAAATTTCTGATTATGAATATCCGTAAAATTTGTGAAAAAGAAAAAGACCAACATCAGTCTATTTTATTAAAAAAATAAAAAGAAAAAATTAAAAAATAAAAATAAAAATTAATTTTGATTTATCTTTTGGTGAATTGACTATATTTAAATTTTTTTTTTTTTATTTTTAAAACTTCAATTTACTTCTATCTTTGTTTAAAAGATAAAAGAAAAATGAAAAGTAATTTGAAAATAATCAATTTACAAAAAAAATTAAAATTAACTCTAAAAATAAGCCTAGAAATGTTAGTTCATTATTTTATAATAAATTAATACTAATAATCATCGCATCCAAAAAAAAAACACTGATAATTATGTAAAACATTTAATGTTTAATTGATTTTCTGATGAATAATAAATCAATAATTGTTCGGTAAGTGAAAAAAAAAAAATACAGATAAGCAACTCAAGGCTGAATCTTCACCAACCCCGTTCTTCACAAATCACCCCAACAACTAAAATACCAAGTTGTTCAGTCAATGATACTCGCATAGTCCACGCCACCACGGTGTTGACCCTTAGATAAAATCAGAATGAACGGCCCTGATCAATCTCCAACCAACCCCTCAATTTCCCACTGCCATTCTTAAGCTTACCTACCAACCCATTATTGATCCTACTACTACTATATAAGACCCAACTCCATGTCTCTTGTCTCCTCAGGAAATCCATTATAGTTATAGACTTCAGAATTTCCTTTAGCTCATTCATTCTTCATCACTCCCATTCATTCATTCTTCTTCTTCTTCTTCTTTCTACAGTTTTGGTATTTTTTGTAATTGCAGTAACATTTACGTAAAACGATATG

>CgPAL2_Pro

CAACAAACATCCACAAGCCACACCCTTCCCCAACTCTTTCAAAAGATATTATCACCTGAGGCAGATCAACCTCAAGCCCCTATTGGAGAAAATAATTTCAAAAAAATTAAAAGAAAGTTCATTGAGTTACTGATTAGCCTCCATCAAATCTATAAAAGTAGCCCAATTAAATTTATAAAACTAATCCCATAAACTCATAAATCCATAAAATTTAAACTTTGACTTTGAGAATGACAAAAGACTACAGTTTAATTAAAAAAATAAAAGCTCATAATAACTCATATGGGTAATTTTACTATTATTTAATTTAACTCACATTAAATCTACTAAAATAACATCTACAAACTCATAAAATTTTATTTTTCACATCTTATAGTTATGTTATTTCGCATCCTGGTAAATGGTGGCACATTGATGTTTGTCAGTAGTACTGGGTTTAGTTTGAAATTTACTCATAAATTTTTAAAATACTTACCATTTCTTTAAGATCCAAAAATCGTGCCACTAAAATATTTTGTCCCATCTATTTCCCCCAAATAATCCACGTGCAAACGAGCTCATACAATTTCTAATTTCTTATTTTAATGGTCGTTATAATTAAATTTTTTAAAAAACTTGATCAGCTACAATCTGGCAGCACTCTCATTTTGACTACGTGCATGTTCTCTGAAAATATTCCGAAAACGAAGTCACATTAATCTATTAATTTATTATTGTGGCAAGATTTGCTACGTAAACTGTTTAAGTCAATAGTCAGTTCATGCGGATCACTTGAGAATAATTTATGTTACTCAAGTCTTAAAATATCAGTAAAATTGTACTTCATTCGAAGATACAACAAACAAGAACATCACTTCAATTTCAACGTACATTAATTTAACTAATTTTTTTAAAATGGCTGTTAGATTTGGGAAAATTTAAACATGTAACGAGGATCTCGAGTTCACATTAAGTTAAAAGCACTTTAAGCCCCTCATTGTTGAATCTCCACCAACCGCATTCTTATTTTCCATCAGCAGTCATCACCAGCCCACCCCCAATTATGGCTGCGAGTCAACATTATTATTTTTTCTCCATCCAACAAATCATTGGCACGGCACACCAATTAATCTTGACCCTCAGATGAAATCAGAATGAACGGCCGCGATCGGTCTCCAACAAACTCCGCATTCTTCCACTGCCATGCTCAGACTTACCTACCAAGTCATTCTTAATCCTGCTACTCCTATATAAATCCCGATACCGTGTCTCTCATTTCCTCAGGAATCCACTACAATTAAAGACTTTAGGGTTCCCTTTAGTTGATCTACTTTCATACACGCCCATTCATTATTCTTCTAGTTGGGGTATCTTTTGTGATTGTAGTAACATTTACGTAAAAGATATGGACAGAGGTGCTGTTATTGAGAATGGTCACCAGAACGGTTGCTTGGAGGGTTTGTGTAAGGACAACAATTACAGTTCTGGGGATGCGTTGAACTGGGGAGTGATG

>PAL3_Pro

TGGTTATGTTCTCCAATTGAAATCATTGATTCCAACCTGCTACTAATTATTAAAATATCAAGACAGTTACAGATCAAGAATTTTAATCTAAAAACAGAATTTATTCAAGTCCTATCTAATTGCCCACTCAAATAAAATAAGAATAAAATCATAAACATTAATTATAGAAGAACATAAATAATCTCAATTAAATAAATTGAATGATAAAAATTGAATATTAATTATATAACATATTTAGGGTTTTGAATTCACCCTCAACTAAATAGAAAATCTAGCGGATCGGGATCCTCTCTTGATCTGAGCACTCCTGATCTTTTACAGATTCTTGTATAACCTGTGTTTTAGTGTTAGTGAATGGTTATGATTTAACTACTTTTTCTTTTTAATTTATTAACCACCAATCATATTAACAGAATTTGTTCAGATGAAGATCAGATCAACAGAGGATCCTGATCCAAATCGAACCTAATACAATTGAATTAAGAATAAAAGAGTTGATAAAACTAGTTATGAAAATTAATTTAAAAGACTTACTCTACTTTTATTTTTTCTTCTCTTCCTGCGATTGTAATCTCCAATTCTCCTATTGGCCGCCAATTGTGTGTTTCTTAAAAAGCCAGAGGTTGGCTTCTATAATTGTTGGTGGAGGACTTATTTGGTACCCTTTGCCTCCTCCAAATCCATGATCAACAAGAAAATTAATGCCTTCCACATGCCATCCACTTTGATTTCAAATCAAAGCCATTTAATTGCCATTGTTCCTCACGTGATTAAATATGACAATTTTCTTTTTCTTCAATTTGCCTTCCATATCATATATAGTCATGCATGTGCAATTGCCAATTCCAATTCTTTATTTGCTAATGAATGTTCGCCGAATGCCGGCTTGCCACGGCTTCTTGACTTTATTCCCCTTATCTTTCAATTGGTTTCCAATTTAATATTGATTGCTTCCCAAATAAAATTTTTTTATACCTTTTTTTATCTATAATCTCTAATTAATTTCCTATCAAATAAAATAATATAATTAACTAGCAACTATAAATTAAGAATAAAATCTACAATAATAATAATAAATTTAAGACAAAAATTAACATAATAACATATAATTTAATAGAATAACTAAATTAAAATTAACTAAAATAATTAGAATATTTAAGACCAAAATAAGAAAAAATTAAAGAAATTAATAGTAAAATATAGAGAAAATATGCACTTATCATATATAATGACTCAATCTGAAATAATAGGCTAGATTCCATTGGAAGAACACAATTATTTGAACTTGTTTAGCATTTTAACGAGACAACTTGTGCTCCACTGAAAATTTCCATAACAGAATTTTATTAAAATTGAATAAATGTCAGCAAAATTTATTAAATTAACAAAATGTCAGAAAAATTAAAATCTGTGGGCCATAAAGTTATTACAGGGCTATTCCGGGGAGTATAAAGTAATTTTCCACAAAATTCTGGCCCCAGAAAATAATGAGGCGACATAGATAGACAGACATTAACCCGCCTGTGAGCGTGCACCAGACACCCCAACAACCTCCATCCTCCTCCAACATCGACGGGTTAAAACACCCATCATGCATCTCTCATTGCCACGTCCACGTGTCATCATCACATCCGTAGGATATCCATGAAGATCCAACGGCCACCGTTCTATTCCAACTAACACCCCTAGCTTCCCACCCAACACTTTCTGATATACGTACCCCCTCAACCTTTCTCTTCTATTTAAACAAATCTTTCTTAAGCTGGTGAACCAACCCCCATGATTTTATTTTTATTCGCTTAGCTTAGCTTACAAAAAAAAAATTCCCCTTTTCTTTCCTAGAAACCAAACAGCCGCGGAACTTACAAATGTCCGTTTGGTTTCTGTGCGCTCTCTCTTTATTGTTTCAAGGCTTTTGAAATAATTATCTTTTTTGGTCGCTACACATATCGTTTTGGACCCGTTAAAGGAAATATTCATTCCTTGGTAACATCTAAACTATG

>PAL4_Pro

TGGTTATGTTCTCCAATTGAAATCATTGATTCCAACCTGCTACTAATTATTAAAATATCAAGACAGTTACAGATCAAGAATTTTAATCTAAAAACAGAATTTATTCAAGTCCTATCTAATTGCCCACTCAAATAAAATAAGAATAAAATCATAAACATTAATTATAGAAGAACATAAATAATCTCAATTAAATAAATTGAATGATAAAAATTGAATATTAATTATATAACATATTTAGGGTTTTGAATTCACCCTCAACTAAATAGAAAATCTAGCGGATCGGGATCCTCTCTTGATCTGAGCACTCCTGATCTTTTACAGATTCTTGTATAACCTGTGTTTTAGTGTTAGTGAATGGTTATGATTTAACTACTTTTTCTTTTTAATTTATTAACCACCAATCATATTAACAGAATTTGTTCAGATGAAGATCAGATCAACAGAGGATCCTGATCCAAATCGAACCTAATACAATTGAATTAAGAATAAAAGAGTTGATAAAACTAGTTATGAAAATTAATTTAAAAGACTTACTCTACTTTTATTTTTTCTTCTCTTCCTGCGATTGTAATCTCCAATTCTCCTATTGGCCGCCAATTGTGTGTTTCTTAAAAAGCCAGAGGTTGGCTTCTATAATTGTTGGTGGAGGACTTATTTGGTACCCTTTGCCTCCTCCAAATCCATGATCAACAAGAAAATTAATGCCTTCCACATGCCATCCACTTTGATTTCAAATCAAAGCCATTTAATTGCCATTGTTCCTCACGTGATTAAATATGACAATTTTCTTTTTCTTCAATTTGCCTTCCATATCATATATAGTCATGCATGTGCAATTGCCAATTCCAATTCTTTATTTGCTAATGAATGTTCGCCGAATGCCGGCTTGCCACGGCTTCTTGACTTTATTCCCCTTATCTTTCAATTGGTTTCCAATTTAATATTGATTGCTTCCCAAATAAAATTTTTTTATACCTTTTTTTATCTATAATCTCTAATTAATTTCCTATCAAATAAAATAATATAATTAACTAGCAACTATAAATTAAGAATAAAATCTACAATAATAATAATAAATTTAAGACAAAAATTAACATAATAACATATAATTTAATAGAATAACTAAATTAAAATTAACTAAAATAATTAGAATATTTAAGACCAAAATAAGAAAAAATTAAAGAAATTAATAGTAAAATATAGAGAAAATATGCACTTATCATATATAATGACTCAATCTGAAATAATAGGCTAGATTCCATTGGAAGAACACAATTATTTGAACTTGTTTAGCATTTTAACGAGACAACTTGTGCTCCACTGAAAATTTCCATAACAGAATTTTATTAAAATTGAATAAATGTCAGCAAAATTTATTAAATTAACAAAATGTCAGAAAAATTAAAATCTGTGGGCCATAAAGTTATTACAGGGCTATTCCGGGGAGTATAAAGTAATTTTCCACAAAATTCTGGCCCCAGAAAATAATGAGGCGACATAGATAGACAGACATTAACCCGCCTGTGAGCGTGCACCAGACACCCCAACAACCTCCATCCTCCTCCAACATCGACGGGTTAAAACACCCATCATGCATCTCTCATTGCCACGTCCACGTGTCATCATCACATCCGTAGGATATCCATGAAGATCCAACGGCCACCGTTCTATTCCAACTAACACCCCTAGCTTCCCACCCAACACTTTCTGATATACGTACCCCCTCAACCTTTCTCTTCTATTTAAACAAATCTTTCTTAAGCTGGTGAACCAACCCCCATGATTTTATTTTTATTCGCTTAGCTTAGCTTACAAAAAAAAAATTCCCCTTTTCTTTCCTAGAAACCAAACAGCCGCGGAACTTACAAATGTCCGTTTGGTTTCTGTGCGCTCTCTCTTTATTGTTTCAAGGCTTTTGAAATAATTATCTTTTTTGGTCGCTACACATATCGTTTTGGACCCGTTAAAGGAAATATTCATTCCTTGGTAACATCTAAACTATG

>PAL5_Pro

CATAAAAGACTACGAAAATGTAAGTACAATTCAGCTCAAGCTCAAGCTCAACTTTAGATTCAAATTATTAACTAAAACTTCGTTGCCACAAATAATCAAACACAATTTATCTGAAACTCATGATTTGGTGCATACTTTAAATCTTGTTTTAGATTTCACAATTATTAGAAAGTATATATTAAATCTTTCAATCAAATTATTTAAAAAAAAAGAAGAAGGAAAAATTCAAGCCAATGAAAGCTAGTGCCGAATGATCGTGCAAAATTCTTTTTGTTTTAAATGTACTTTAGAATTTCAGTTTTTTCGCATATGGTGTGTCTTTATTATTTGACATTTCTAAAACATGATGTGCTTCTTTAATGAATTTTGACCACGATACTACTAGACATCTCTACTTGATTCGAGATATATTACCTTATGTTATAAATTTTTTTATTTTTTATTTTTGGAAAGTTACCTTATAACACAACTAATGCTTATTCTCTCAGCAAATGAACTTCAACGAATTTACTTCTATAAGACTTTTTTTTTTAATAAAAAAAAAAGTAAAAGAGCAGAAAAAAAAACTAATCATTTCTATTTTTAATCTCATATTTTTTTTAGGATTCGAATTCAGGTAGCCAGTTGGCTAGTCCAATTAAACCAAATACAATTATTCAAAATCACTCTGATTAAACCCTTAAGAGGACCTCTACATAAATATATACTAAACAACATGCTGTCTATTATATAAAGGCTCTTACAGCGTACGTGTTTATTGTTTAATTGCATAAGAGTGACAAAATTTAACCCATTTGCAATAAAAGGGAGCTTAACTTTTCAATATAAACCAATTACACCTAAACCATTTCATCCAAAGTCCAAACACCTCACTTAATGGGAGTCCGCGCTAGTCTCGATAGAATTTTGGGCTGTATTATAAATTTATATATGTAGATTCTTAATAGATTGATATCAAATTATAGTATATTTCTAAATTGTAGCAATTTAAAAAAAAAAACACACACACCCCCTTACTTCATGTGGTAAAATTTAAACCAAAAGCAAATTAAGTAAATGATCATTGCTTATAATTGAAAAATGTACGTGGATGTTGTAACTCGTAATATGCCAAACAGATAAAAATTGAGTGATGTTGCCATTGGAGACATTAATTCTTTTTCTAAATTTAACAATGAAATGGGTATCCATACCCATAAAATTGCCATTTCTTACACTACTCGACTCGACATATATGAGATATTTTTCCTTATCATACA

ATAAATGCTTATCCTCTCTATAAATGAAATTCAATGATTTTCCTTCTACTAGAATATCGAAATAATACAAAATAATTTGTAACGTGCATAATGGTATAGAGTGTCCATTGGTGTGAAAAGAGGGACCTTGATTTTTAAATATAAATCAATTATAACTCAGCCATTTCATCGAAACACCTCATTCATTTGATGTTGCAAAATCTAATGCAATTTTCCAAACCCAGCAGTCAAAAAACGAGTGAAGTAAATGACCATGCAATTGCTTAAATGGATACTTAAAAAGTATTCATTCATTTTGGAACCAATTTTGGGGCTTTGGTGAATATGAGGTATAGAAATAAATTGGATTGGGGGGGTGGTGACGTTGTGCTTTCGTGCCCCCTCACCAACCCCGAAAAAGTCCATGCCACCAATCACCTTGCTTCCATGTGGCGAGATCTTAACCATCTAAAACCATGAAAATCCAACGGCCGCGTGTTTCTTTCAACTAACCCGTCGGCCAACCCCACCAAACACTCAATGTTGCCACCCCCCCAACTCTATTTAAAGCCCCCGTTTTAGGTCTCCAAACTCAGGAATTTTCACTTGGCTTGAAAATTTCCCTTTCATCATCGTCACAGATTCACGTTTACATGCAATAAATATATAATTGCCCCCACAAAAGATTTTCCCACCCATTTTCTCTCCCACCCATCAGTACATTTACTTCTTTTAACTAAAAAACAACAAGGAAAAAAAAAATG

>Cg4CL1_Pro

ATTGTATATGGTTCCACCTCTATGGGTGGTGTTAAAAGTACCATTTGAAGATATTAGAAATCCATCTTATCCCAATATTAATGATATGACTCAAAAATAGTTGATTTTGAGGTCGAATCTCAGGGAATTCAGAAAACTAAACAGTCAATATTATTTTTCATAAATTTTATTTTGATTTTATACAAAATCTAAAAAAATTTAATTGATTCTCTTGGTAGTGGGTTCTCTGTATGAATATTAATCTTCAATTAAACTAAAAAAACATATATTTGCAATAACAATATCTTGTTAAATTTATTGCTGAAAATTATTGGATTTCAATTTTTTAAAGAAGTTATTTTATATAGGTAAAAAAAATAAATACTTAAATTATTATAGTACAATAACACACATTTTGTAATTAATTTGATCCTCTTGTATTATAATGGTGAAATAGTCAAATTTCATTTACGTTAGAGTAGTCTCGAAGTCAAAGATGGATTTTAATTGATTGATTATGCAAACTACATTTTTATACTAAATAATACAAATAAAGTGATGACTTTTGTACGTGTGTGTATAATAGAGTGGCCGTCCCCAAGGCTTGCGGATGGGTTAGGCTTATGCCCAGTGCCCACCAACAACTCGACCCGACCCATAAGCCTTACCACGGCCCTTATGACTAATCCACAAAGTTCGGGTAATCACACTTGCCATGCAGGACTAAAGGGACTTCAAATCAAGCAAATTTTGAAAGCACAACAATAAAAACGTTTATTTTACACTCACTGAGTCACTGTTATTTGACCCAACTAATTCTAATAATTTTTTTTATACTTTTATTTATTGATCAGTTATTTCATTCCACAATACGGACGTAAACCCGTAAAACTGCGCGGTGATTTGACCCGATCCAACCCATTTGACAATGATTTGGTAGTAGGTAATGGTCTGATAATTACATCTGAGGGTGTGGTAGGTGAAAAGCTAAGAGAACTTGAATATGAACGCGAGGCCACAGGCCCCAGGTCAACTTTGCCAAATCTACACCACCAAACCGTGGACCCCACTCACCACCCAAACCTATTCTAACCCACCAACCCAACTCCACATGACCTTCCGTCACCATGCCATCACCAAACCCATTTGCACACATATTTTATCACACTCGTATTCTCTCACGTTTGCCCTCGAAACTCTTTCACAATTCCCATAATACCCCCTCACCAACTTTCTTCACCAACTTCAACACCCAATGCTGCCTCTATATCAATATCAAACACTTGCCGTTTCCCCTCAGAAGCTAAAAAAATATACATATG

>C3H_Pro

AGATTCCAATGGTCTGCGTGTATCTCTCAATCTCACCAACCCACCACCAACCGCAATGGGTCCCGTAATTGATAATACTCGGATACCCTTTTTTAATCTGGATTAGACACCTCCCAAATCCCAAGCCACCCAACTTACGCCAAATTCACCACCACCCAAACAAGGAGCAACAACAACCTACTACAAATTATCTTGCCAAAAACCAAATCTTTAATGACTATACCCAAGAGTTTTAAATATTTTTACTTTAAAAAAGAAAAAAAATTTTAAAATTTTTAATTTCATAATACTGTTGATGTTTAAGAAAAATCTTTAAATACTCAAATAAATATTTATAGAGGGAAAACCTTGATTCAAATCGTTTATATATACATATATATAAAAGAAACAATATTTATCATCATTAACATACAATTTAACACAACTAAAGCTTAGGAACATTTGTGGTGATTCCTGATCCAGAAATTGAATATAGATTACAGTGCATATTTAACTTATTGGAAATGGAAACCATGGATACCACCAAAATATATACAGTCAACATCATTGTAGCATATATAACTTTGAAATATAGTCAATTTTTTTACCTAACATCTACGCTTAAATTTCGTTTCGTAGAGCATAGCTTAAGAAAGAAGGCAAGAAGCCAAGAAGCAGTCGTGATAACTTCTTCCCCATTTAAGTGCAACTATAATTTCAATCATTATTACAAGGACAAAACTCATGTGTAATCATGCGAACTCTTAGCAGTCGATGACAGGACATGGCTCCATACAACTTGATTACCGCACACGTTGCTTCAAAATGAATTACATCTATTTCTTCTTTCGGTGGACTGTACAGTGGTTTCACTTTTTTTTAAAAAAAATTATATTAGACTAATGCTCATATTACAACTTATATTATACGATATTTATAACTGATATTTATAAATCAGACAATTACTAAAAACTAATTTACATTAATTTTTATATAATGATATCTAAATTTTACCCTCTCAAATACGAAGAGTGTATACTTCACTCACATTTAAAAAAAAAAAAAGTATGATTTCACTTAATTATATACTATAATTAAGAAAAGAAGAAACATCTACATGCATCGCGAAGGCTTGAACCGTTACTCTCATAATTGTGAGTACAGAGTCTTGGCCATTGGACCAATATAGTGGTTTCACTTTCATTTCATTATCTCTCCTTTTTTTTCTCTCTTTTAATTCATATTTTTCTGACTAGGATTACATTGACATTGACAGACCCTCCCACCCTTTTCATGCCATCCAAGGATGCATTATTGTTCTAATTTAATATATTTAATATAATATTTTTTATCTCACAGCTTGAATACCGTGAAATAATTAATTTTTATAAACTTTCTAATCATTTAAGGCTGCTCTTATAACCCTTAAAAATAGCGTAGTTGGACTCTACAATGTATTATGGTTCGTTTTCATTCATTTGAATTTTCATCAATCCGTTGAGACACATCAAACGCGTACAAATGAATTCGTGGGTTCATCAGAGCAGTAAGAGTCTAACACCTCACCAACCTTTGAATCCTCTCCCGCACTCCTGTTTCCAAAAGTTTCTCACTTCACCAACCCCAATTTACAAAATGCACTAATCAAAAACAATCCCCACCCTCTCAATTTCGAAACTATCATAACACGCTCCTTATGATTGGCCCGATTCATGTGAGTCGGCCAACCATTAAAAACCATATGCTTGGGCGTGCCATTCATCAATTCTCATTCATGGCAACCATTCATTTCATGCTTTAGCTAGTGAGTGGTGACTTAAATTTTAGTTTCCCACGATATAATTATTATTTTTCTTTTATCTTTTTTATTTACATGACGTAATTTCTTAACAACGTAAGCAAATTCATCCACCAACTCCCCCGTTCTTCATCTCATATATGTCTCTAAGTCTATTGGTCTAACTCAAGCCCAACCATTTCATTTCCGAAGTTTCAAGAAAAAGGAAACCAACCAACACAAAATG

>CCoAOMT1_Pro

ATGCCTTTATGAACTATTTCAAAGTAATTTAATCTAATTTTAAAATTAATTTATAAGGGAATAAGAATATTAAAAAGTTGCTGGAAAAGGATGGGTTACAGTGAGGGCCCGCAGCCAGCTGTTAAGTCTCCAAAGCAGGCCGACATAATTTGACTAATTTTTAGGATAATTTTTTTTATTAAATTTAAGGATATGTTCAACTTACTGAATTACTAAATTAACCTAACTTTTAGTCTTAATATTTTAAAATTATTAGTCTTCTTTTTTTCAGAAGTTTAGGTTGGACTAATTCTCAAATTTTAATATAAACTAAATTTGATTGTATCAATAATGAATTTATTTTCAATAATAATTTTCATTTTATTTAATTTAAGATTGAAGGCTCTATTTGGGACTAAGGGTCTATATGATATTATTTTTGGGAGGCTTAAAAGTGATTTAAAAAATTTAAAAGTTAATTTTGGTGTTTGGTTAAAAAAAAAAATCACTTTTGTCAAAATCAGCCCCTTCCACATATGATTTTGAAAAAAAATGAATGAGTAACTTTAAATTCTGATTTTGAGAATCAATTTTATCATTTAATATAATTCAATAAATATCCTTGTAATTATTATCTAAACACAAAATTATCCTTATTTAAATTGGAAACAAAATCAATATTTAAAAAAAATTAATTGTAATTCATCTATTCCTTTCATTTATCCTATTTCTCTTTATATTCCACATTACATCAAAAGTTCTCTATGGCAATATAGTCTAAATTTTATTTTTAGTTAAAATTATTATAATACACAATTAAAATCAATATAGTATAAATTTTATTTTAGTTAAAATTATTATGCTACACAATTAAAAATATTGTATATACATATTTTTATATGTGTACGTCAATTTTATCATTCATTATGTACATCTTAGTCATTTGTTTTCTCACAACAGTTTAACAGTAAAATTTATCAAATAGAAATAACTGCTTTTAAAACTCACAATACTTTTAAAAACAAAATTTACCAAACATTTAATTGATTATAACAGTAAAATTTATCAAATAGAAATAACTACTTTTAAAACTCACAATACTTTTGAAAACAAAATTTACCAAACATTTAATTGATTATTTTCACAATTGATTATTTCTACAACACAGCTAACAACAATTATTTTAAAAGTTACAGCATTCCCAAACTGACCCTAAGATTGAAATTTTATAAACTGCTTGTTTTATGATTTTCTTCATTTTGTCCATAACTTTTAAAAGTAAATAAAAAGTTATTTTTTATAAACAGCTTATTAAATAAGTTACTGCACCTTTTTAAAATTTCTATTACAACCTTATTATTTAAGAAATAGACAATTATATTTGATTTTTTCATAGGTCTTGATATATAAACCATTATATATCATGTTACTACAAAAACTAATGTGCATTTTCTCAAGTATCAAAATTGATCCAGTAAGTTTATATGTATATTGTATTTTATGTTTATTTTCTATCATAAGAGTCAATAAGATTTTCATATAAATGTAATAAATGCTATTTTTTTTATTTTATTTGGATGTATATATTAGTATGATTTTTGTCATATTTTATGTAATATGACAAAAAGTATGTCATTTAATTGAATTTTTTCACTTTATGTAATTTTACATATTTTAAAAAAAAGGTATGTCTAAATAATTTTATTAAATTTTTTTAAGAAGCAAAAGTTACTTTAAATTTATATTTATATGATCATTATATAATAAAACAATACTTTAATAAAATTTACTAAATGTAATTTATTTTTCCAACTTATCGCAACAAATACAATTTACTAAATATCTTTACTTTTTTTTAATCATCCGCACATGCTTAAGATTGTACTTTTTTTTCTTTGGTCTGAAAAAGTAGTCCAATCCTACTTATGGTCATAGCCTCCAACATGGCATATTATTAGTGGAGTAGATAATATAAAAGGAGGAGTAAGGAAGAATTAGCACCATATGGTATTCGGTAAGTAATG

>CCoAOMT2_Pro

GTCACTTTCCTAGGCCTCGTCGTCTTAGGAATCAAAGCAATATATATGTGATTGATGAAATACTAACTAGGCAACCTAAGAGGGGGGTGAATTGGGATTTTAAAATTTAAACTAAACAAACCACACAACAATTCAATCTAATGCTTTAATGGAATCGAAAACAATAAATTCAATAAATCACAATAATAAAAGAGTAAGGGAAGAGAAAAAAAACACAAGGATTTTTACGTGGTTCGGCAATCCCTGCCTACATCCACGCCTCCAAGCACACCGGGCTTAAGGATTTTATTATTCAAGCCTCCTTGCCATGCTCTGCACTTCTTGCTTCTTCCTCTTCCTGCTTTTTGACTGAAAAATACGAAGACCAAATAGTTCCCTCGACATTGTTCAGAGCTTTCATTCTTTTACATTTCGGGCTTCTTTTTTCTTGTGTTTCTATGGGCCTTTTATGTGATATTGGTCCATTGTTCCGGCTCTCTGCAATTAAACTTCCCCGTGCCTGTGATTTCTATTTTTTTATTTTCCTAATTTTGCCTCCCACAGACACTTCCATTCCTTTATTCTTGGTTGTCTGGCCCATGTCATTTTCCACTTCTTCGCAGCCCACTAAGTCCATTTTTCCCGTTTCATTTTCCATCCCGTTTTCCCGCCTTTCTTGCTTGTTGTTTCCCGCTATAGTGCTCATTTCTCTAGGAAATCTCTATGCATCTGTTATCTCTGCTACTTTGCTGCCCTCTGTAGCTAGTTGTTCCTCACCCTTCCCAGTGTGCATTAATGATCCTTCATGTAGCCCTTCCATGTGGTCAACCCTCATTGGCTCTGTGCTAGGACCCGTAAGAGATCGGGTTAACTCATATCCTCCTTCCAAACCTTGCTTCCCCAGACCCGTTTCTGTTTGCCTCTGTTCTTGCTGTTCTTGTGGTTCATGATTTGTTGTTGCCGATTCTGATTTTTGCTTAGCTTTCCAATAATCTTGGTTTGTTTTCTGCTTCACTCGTTCCATCTTTGTTAGCGCCTTCATTCAAGCTCCATATGGCAGTTCATCCTTTGGCTGCCATTTGCACTCAAGACATTCCTTATACTGATGCCCAAGAAGCCCACAGCAAAAGCAAAAGTCTGGTAACTTTTCATACGCCACCCTTAGCGAGATTTTCTCATCATCTTCTAACCTTAGCACTTAGCAGCAGTCTCTTTTTTAACGGCTGTGTTATGTCTATCCTTATTCTTATTCGAGCAAAGGAGCCTATGCACCCTCCTATTTCATCTGTTTCCACCTCCTCCGCTTTGCCGATCTTCCCCCTATCTCCTGCATTATGTCCTTGTCCATACACATAATAGGCACATTATGGATTTGCACTCATAATGATGTATGAGTGAAGGCTTGCTGTTTAATATTCCCAACACCAGTTGGCTCCACAAGCACCATTAACAAATTGTTAAAATGACATGGTCCCCCATGTAGTATTCTTTTCTTTTCTTCCTCTGAAGCGAATTTGAAAATAAAAATATTTTCTCCTAAGCTCTCCACTTTCACTCCTTTAACCGATCTCCAAACTTGCTGCATGGCTGCTCTAAGCCTTTCACGAGAGACTCCCCGTGTATGAAGAATTTTTCTAAGGAGGCAATGTGCTGCTATTTTTGCCCCCTTTGCTTTCATTTTCCCTGCAAAGTTAATCATATTTTCTTCTTCTGACTTTAAACTTATGGCCGCGCATTTTCGTATGAGTTCGTCCGAATCCATCTCTCCCCTTAAAGTCTACTAGTTTGCACTGATGATCCTCACCAACCTCCTGCCAACCCAGTTATCTGTAATAACTGACTTCTTCAACTTAATATCTTTTCTTGTATCTTTCTTTAGTTCCGACCGTGTATGCCACCCTTCTGTCAATGGCACGAACCACTCAACCCGTGAGTGACACTATTGGATTACCAGCGAATCCTGTTTTCCATCCATTCTAAGTTCTCTTTGGGAGAGAGCTACCTAGAAGGAGAAGCCAGTGATG

> CCoAOMT4_Pro

TTTTTTTTTTATCAAAAGCTGTTTCTTTGTTAATTGGAAGCAAACGTCAGAGCAGTATAGGAGAGTGAAAGCCAAAATGAAGAGAGAAAGAAGAAATAAATGTAAGTGACTGAAAAAGGAAAAAAGAAAAAGAAAAAGAAGAAGAGTGAAGACAAGCCGGAGAAATCTACATACCCAAGATCCCCATCAAGCTCGTGAGTGAATTGCTTTGTTACTAATATACACATTCCATGAAATAAATATAAATAATAGTATGGCCTAGTAATACGTTATGATTAGATATGTGTATGTTATATAATTAATCATGTCATGAGATGTAATTGTGAATCACCAAATAAGTACCATTCTCATTAGGTTTTTATTATTTTGTACATACATATTAACATGTGTTATTTAATTTATCAGTGTGATAATGCTAGTTTCGTAAGCACAACATGATTAAGTGGTTATCATGTATTATACTTAGTATAAAGTTCGTGTGACATGATTATTTCCTATGATTTATGCATGCCACTATTTAGATTATAAGCACCCACCATATATATTAGTCAAAGATTCACATATTACTATCTATTTAGTTACATTCATATCGAGAGTTCTTTAAATCCAATTCAATTTAATCAATAGTTCTAATAAAATCATGCACCATCATACTTATAAATTCGTCCGTGTATCAACCTCGCAGAAACCCTTACAAACACTCAAAAAAAAAAAAAACAATATGAATAACAATGTAGTTTAATAACATGTTATGATTAAATGTGTGCATGTTATATGATTGATTATGCCAAGAAACACAGTAACAAATTACCATATGTGTATCCATGATCTTCTGTATTAAGTATTCTATACGTGCATACTAATTTTACTTTGTCAGTGGGATGCTGTTAGTTTTATCAGGGATGACATGTTATATTCACTCTGCATCTGTGTGATGATAGTCCCGCGCGAGCATGACATGTATTTTCTCAAATGCTATTTGATGTAGCTTTTGACATATTTGCCTTGTGATTGCACGGTTACTCATATGTGAGCGTGAAGACTATGTTATCTAATCTTTTCCAATGGTTTATATATGCTGCTGTTTAAATCATAAGCACCCACCAAATTAATCATAGATTCAAATTTTAGTATCCATTAGTTATACTCATAGATTCAAATTCTAATCAATAGTTCGAATAAAAATCACACACCATCGTATCTCACTCAACTGAGCAAATCTCTAGTAAATACCCAAATAATCACTAATAATAAGACCAACTGTCAAATTGAGATTTTACTGAACAGTCCCCCTTTCTTATTTTTTAAGGAAAAGAGTTGTATTATCTAAAAGACATGAAAAGCGTCACTCTTATTTGTCAAGAAATGTGAGGTGGCCACCAACTCTGCTTTCTGCATATACCGAACCAACATGGGCCATGTGTGAAAATTCTAATCTTCTCTTCCCTTCACCAACCCTTTACCAACTTTGCTTAGCCACATTTTTTTTGTTTATTCTAATTACAAAATAATATGCCCCCCCACCACCCTCAAAGAGAGCCTAATCTTCTAGCAAACGTACCTACAATCACCTAATTACCCCCAAGGTAATCTCATAACGATATACATAAAGATAGTTAAATCCCAACTTAAATAAACAAAGAGGACGACTAAAATTTTGAAATATGCTAAGAATTTATGTTGATTAAAGAAATGAAAACATTTAATTGACGAGAAAATAAGTCTAACGTTAGCTACAATTATTATTATAAGAGAGAGTGATGAAATGATGATGAAAAAAATGTTGGGGAACAAATTTTTGAGCGTGGGCCACGAACAAAAGACTCATGCTGTCTAACTTTGGACTTCACCAACCCCCGACCGGGTTTTCCATCCAGCCCGGGCAAGCAAACCGGCCTCTCAATTTCCCATATAAACCCCATCACAGCCTTCAGATTTTCCAACTCAAGTTACTCTTTTAAAACTCCAAAATCCAACATCAAAAGAAAAGACTTGCGTCAATG

Note: AC elements were highlighted with yellow, ABA response elements were highlighted with purple, auxin response element was highlighted with green and the start codon were highlighted with blue.
